# Facile and Reliable Emission‐Based Nanomolar Anion Sensing by Luminescent Iridium Receptors Featuring Chelating Halogen‐Bonding Sites

**DOI:** 10.1002/chem.202002738

**Published:** 2020-10-07

**Authors:** Robin Kampes, Ronny Tepper, Helmar Görls, Peter Bellstedt, Michael Jäger, Ulrich S. Schubert

**Affiliations:** ^1^ Laboratory of Organic and Macromolecular Chemistry (IOMC) Friedrich Schiller University Jena Humboldtstrasse 10 07743 Jena Germany; ^2^ Jena Center of Soft Matter (JCSM) Friedrich Schiller University Jena Philosophenweg 7 07743 Jena Germany; ^3^ Laboratory of Inorganic and Analytical Chemistry Friedrich Schiller University Jena Humboldtstrasse 8 07743 Jena Germany; ^4^ Center for Energy and Environmental Chemistry Jena (CEEC Jena) Friedrich Schiller University Jena Philosophenweg 7a 07743 Jena Germany; ^5^ Current address: Intelligent fluids GmbH Karl-Heine-Strasse 99 04229 Leipzig Germany

**Keywords:** anions, density functional calculations, luminescence, noncovalent interactions, receptors

## Abstract

An anion sensor is presented that combines a bidentate hydrogen‐ (HB) or halogen‐bonding (XB) site with a luminescent monocationic Ir fragment for strong binding of common anions (*K*_a_ up to 6×10^4^ m ^−1^) with diagnostic emission changes. A new emission‐based protocol for fast and reliable detection was derived on the basis of correction for systematic but unspecific background effects. Such a simple correction routine circumvents the hitherto practical limitations of systematic emission‐based analysis of anion binding with validated open‐source software (BindFit). The anticipated order of *K*_a_ values was obeyed according to size and basicity of the anions (Cl>Br=OAc) as well as the donor atom of the receptor (XB: 6×10^4^ m ^−1^ > HB: 5×10^3^ m ^−1^), and led to submicromolar limits of detection within minutes. The results were further validated by advanced NMR techniques, and corroborated by X‐ray crystallographic data and DFT analysis, which reproduced the structural and electronic features in excellent agreement. The results suggest that corrected emission‐based sensing may become a complementary, reliable, and fast tool to promote the use of XB in various application fields, due to the simple and fast optical determination at high dilution.

## Graphical abstract

**Efficient sensing**: Hydrogen and halogen bond‐based receptors bearing a monocationic Ir‐complex fragment as luminescent probe were synthesized and characterized with respect to their emission increase on addition of common anions. Systematic background effects were considered during analysis and the findings were corroborated by NMR, X‐ray crystallography and DFT calculations.

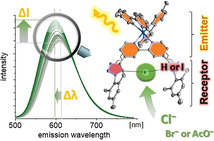

## Introduction

The recognition and sensing of anions have received enormous attention due to their important role in chemical, biochemical, and environmental processes.^[1]^ Sensing by specific molecular receptors relies on the diagnostic change of a detectable signal on binding of the analyte, for example, by NMR spectroscopy, isothermal titration calorimetry (ITC), as well as electrochemical and optical methods.^[2]^ In practice, the limit of detection (LOD) depends on the strength of binding *K*_a_, the induced changes of the diagnostic signal (chemical shift, heat, emission, etc.), and instrumental signal‐to‐noise ratio of the applied method (NMR, ITC, spectroscopy). Consequently, the instrumental methods operate in different concentration regimes, which result in a wide range of requirements to ensure solubility (e.g., specific solvent mixtures), as well as differing data accumulation times and analytical interpretation (vide infra).^[3]^ Herein, we report on the facile luminescence detection of halogen bonding (XB) and the related hydrogen bonding (HB), as detailed by a systematic analysis.

XB is a supramolecular interaction (R−X**⋅⋅⋅**Y) between an electrophilic XB donor site (R−X) and a nucleophilic XB acceptor (Y, e.g., anions). To maximize the strength of the XB interaction, an electron‐withdrawing moiety (R) is used to polarize the halogen atom X. The resulting so‐called σ‐hole^[4]^ is characterized by an electrophilic spot on X located at the far side of the R−X axis.^[5]^ Notably, XB donors have high binding ability (association constant *K*_a_), as demonstrated by cationic imidazolium^[6]^ and halo‐1,2,3‐triazolium^[7]^ groups as well as uncharged halo‐1,2,3‐triazoles^[8]^ and perfluoroiodo arenes.^[9]^ In view of anion recognition, the greater preference of XB for linearity^[10]^ makes XB systems generally superior to HB.^[11]^ To date, HB receptor molecules^[12]^ have been explored intensely,[^1a^, ^13^] whereas the related XB congeners advanced the field more recently,[^1b^, ^10^, ^11^, ^14^] including crystal engineering,^[15]^ organocatalysis,^[16]^ as well as self‐organization and supramolecular templating processes.^[1b]^ Notably, multidentate binding motifs lead to increased *K*_a_ values due to cooperative chelation of the anionic guest.^[6b]^ Hence, the intervening bridge (or spacer) between the donor motifs plays a crucial role in ensuring the optimal cavity for a specific anion.[^6e^, ^8c^]

Among others, Beer et al. extended this concept to design specific cavities by means of supramolecular rotaxane architectures that combine a strong XB receptor and a sensing unit within the interlocked structure.[^1b^, ^7b^, ^8e^, ^17^] This sophisticated yet powerful approach even enabled the order of anion binding affinities in the halide series (Cl, Br, and I), which is conventionally governed by their nucleophilicity, to be reversed.^[8c]^ In addition to the receptor site relying on XB interactions, a (sensitive) reporting unit is necessary for detection. For this task, NMR spectroscopy or ITC analysis is frequently employed, whereas optical changes, in particular emission changes, are much less utilized in a systematic fashion. In this regard, metal complexes are excellent sensors due to their advantageous ^3^MLCT emission properties.^[18]^ For this task, the prototypical complex [Ru(bpy)_3_]^2+^ (bpy=2,2′‐bipyridine) proved to be versatile for HB‐based sensors.[^1a^, ^13a^, ^13c^, ^18b^, ^21^] Beer et al. recently incorporated the [Ru(bpy)_3_]^2+^ complex into XB‐based rotaxanes with a designated cavity size, which led to excellent iodide recognition and sensing even in the presence of water (*K*_a_ up to 10^5^ m ^−1^).^[8c]^ Ghosh et al. utilized a related Ru^II^ complex for phosphate sensing, which exemplified the superiority of XB‐based sensors (*K*_a_ up to 5.6×10^4^ m ^−1^, LOD=18 nm) over the HB analogues (LOD=50 nm).^[22]^ Recently, an additional increase in anion binding by 50 % was reported for the XB sensor when augmented with further HB‐based moieties.^[23]^ Interestingly, alternative anions lead to substantially lower *K*_a_ values, attributed to the exceptional interaction of polyphosphate‐based anions. In their most recent extension of the phosphate‐specific aggregation phenomena, Ghosh et al. incorporated a pyrene signaling unit, leading to a maximized quantum yield of 3 % on aggregation.^[24]^

**Figure 1:**
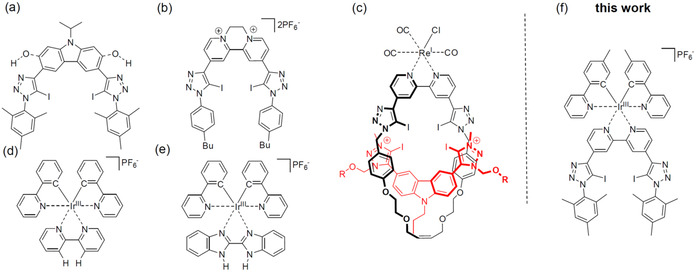
Schematic representation of selected bidentate XB receptors based on a) carbazole^[8d]^ and b) bis‐*N*‐alkylated bipyridine,^[8b]^ d, e) luminescent Ir‐based HB sensors based on d) the bpy^[19]^ and e) benzimidazole ligands,^[20]^ c) a luminescent rotaxane based on a strong dicationic XB receptor subunit (bis‐triazolium fragment shown in red) and a weak XB‐based Re sensor subunit,^[7b]^ and f) an Ir‐based bidentate XB sensor (this work).

Although Ru^II^ complexes are potent sensing units featuring highly beneficial charge assistance of XBs,[^13b^, ^13c^, ^20b^] their twofold cationic charge may induce solubility issues as well as unspecific electrostatic interactions to reach charge neutrality in organic solvent mixtures. Hence, the structurally related but monocationic congener [Ir(bpy)(ppy)_2_]^+^ (ppy=phenylpyridine) is an attractive alternative.^[19]^ Moreover, Ir complexes have comparable or even higher emission quantum yields (typically >10 %),[^18a^, ^19^, ^25^] which make them well suited for 1:1 recognition processes with singly charged guest anions. Surprisingly, this versatile combination has not been exploited in depth, despite scattered reports indicating their general ability to act as HB sensors (Figure 1 d and e).[^19^, ^20^, ^26^] In fact, the level of interpretation of the emission data can be substantially hampered due to the occurrence of additional processes, which are termed “background effects” for simplicity in the following in relation to their impact on the observed emission changes. Notably, a background effect has been identified recently for NMR studies, which was assigned to the ionic strength and thus affected the calculation of the association constants.^[27]^

## Results and Discussion

Herein, we present a systematic study on a bidentate XB‐based receptor with a luminescent Ir fragment (Figure 1 f) for anion sensing. The molecular design descends from our previous bidentate systems,[^7c^, ^8d^, ^28^] whereby the carbazole bridge (Figure 1 a) is replaced by a bpy unit. A similar receptor subunit reported by Beer et al. served, after twofold quaternization, as an electrochemical sensor (Figure 1 b) or, after attachment of a neutral Re‐complex fragment, for incorporation into a dicationic rotaxane structure (Figure 1 c). Interestingly, no analysis of the Re‐complex fragment itself was provided, nor was coordination to typical Ru‐ or Ir‐complex fragments found. Hence, the target structure comprises charge assistance for 1:1 interaction with singly charged anions by the monocationic Ir fragment, preorganization of the bpy scaffold due to complexation, and electronic communication with the triazole binding units through π conjugation.

### Synthesis and characterization

**Scheme 1:**
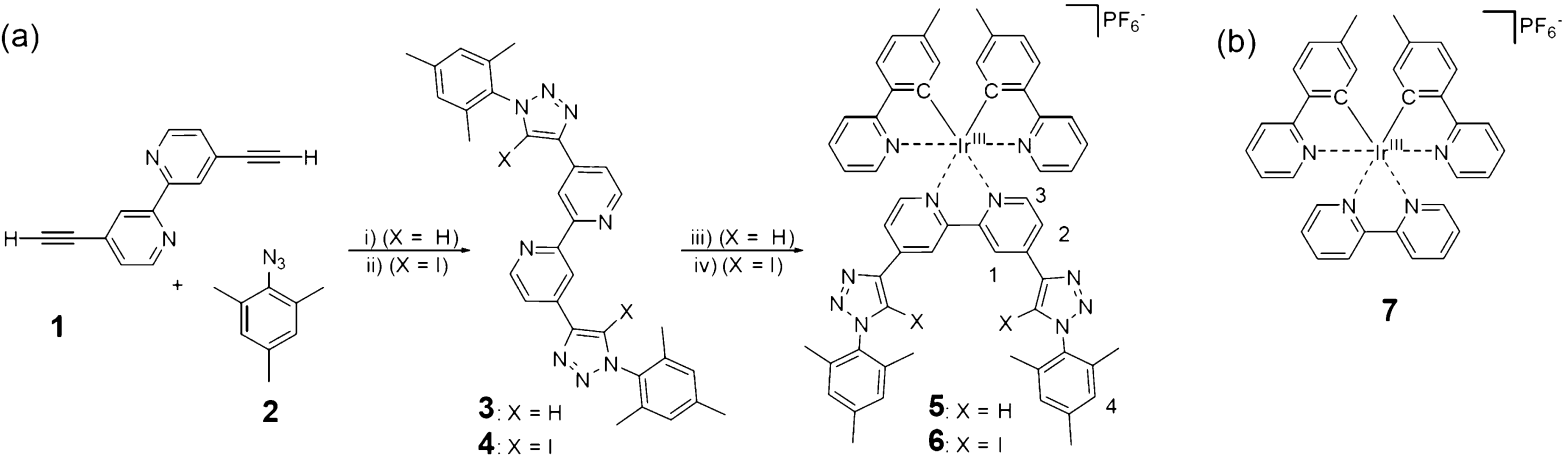
Schematic representation of a) the synthesis of the HB and XB sensors and b) of reference complex **7** without a binding receptor motif. i) CuSO_4_, sodium ascorbate, CH_2_Cl_2_:H_2_O:EtOH (1:1:2), 50 °C, 23 h, 90 %; ii) TBTA, DBU, NaI, Cu(ClO_4_)_2_, THF, RT, overnight, 80 %; iii) and iv) [{Ir(Meppy)_2_Cl}_2_], CH_2_Cl_2_/MeOH, 50 °C, 3 h, 85 %.

Scheme 1 a shows the modular synthetic route towards the XB and HB sensors starting from the common building block **1**, which readily reacted with mesityl azide (**2**) under standard CuAAC conditions[^7c^, ^8d^] to yield the HB receptor ligand **3** in 90 % yield. Similarly, XB receptor ligand **4** was directly prepared in 80 % yield by adjusting to iodinating conditions.[^8b^, ^29^] Notably, the direct iodination during triazole formation is the key step to circumvent the synthetic challenge of the conventional route involving halogenation of triazoles, which requires too‐basic conditions and, thus, is typically incompatible with the bpy fragment. The final coordination step proceeds from the binuclear precursor [{Ir(Meppy)_2_Cl}_2_] [Meppy=2‐(2‐methylphenyl)pyridine] to form HB sensor **5** and XB sensor **6** in 85 % yield, respectively. In analogy, reference complex **7** without a designated receptor subunit was synthesized (Scheme 1 b). More experimental details including analytical characterization and NMR spectra are provided in the Supporting Information (Figures S1–S8).

### X‐ray crystallography

**Figure 2:**
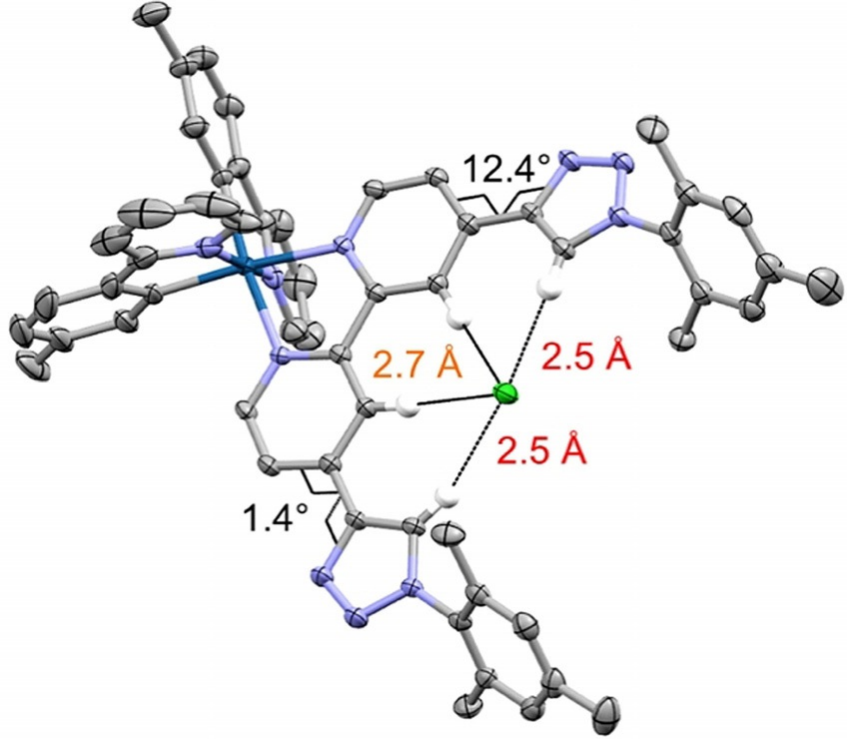
Molecular structure of sensor **5** with chloride anion [thermal ellipsoids at 50 % probability; hydrogen atoms omitted for clarity, except for HB of the bpy subunit (orange) and triazole units (red)]. Gray, carbon; light blue, nitrogen; dark blue, iridium; green, chlorine.

For the neutral HB receptor motif **3**, suitable crystals for X‐ray diffraction analysis were obtained and revealed a *transoid* orientation of the central pyridine rings and intermolecular HB between the pyridine nitrogen atoms and the triazole hydrogen atoms (Figure S9 of the Supporting Information). Crystallizing HB sensor **5** in the presence of chloride counterions lead to XRD‐suitable crystals of **5⋅Cl** (Figure 2). In contrast to the free receptor motif, the Ir‐containing sensor revealed preorganization of the two HB motifs and their bidentate coordination of chloride. The cationic iridium fragment features the typical octahedral coordination geometry. The peripheral triazoles adopt a quasicoplanar conformation with respect to the bpy units, as shown by the minor dihedral twist along the interannular C^bpy^−C^trz^ bond (1.4 and 12.4°). Furthermore, the terminal mesitylene groups are rotated out of plane with respect to the triazole unit. Consequently, the triazole C−H bonds are only marginally displaced from the bpy plane to accommodate the chloride guest. The H^trz^ **⋅⋅⋅**Cl distances are 2.453 and 2.518 Å, respectively. The corresponding C−H^trz^ **⋅⋅⋅**Cl angles are 162.1 and 169.4°, respectively. The asymmetry is tentatively assigned to crystal packing effects. The chloride anion further showed close contact to the bpy hydrogen atoms. Notably, the H^bpy^ **⋅⋅⋅**Cl bond lengths of 2.672 and 2.689 Å are significantly shorter by up to 0.2 Å than those reported for the [Ir(bpy)(ppy)] fragment.^[19]^ This difference is assigned to the cooperative effect of the triazole HBs, and suggests that also a larger *K*_a_ value should be expected (vide infra). As a consequence of the nearby Ir center, the hydrogen atoms had to be fitted by using standard values, so that the experimentally observed C^trz^ **⋅⋅⋅**Cl (3.434(3) Å and 3.391(3) Å) and C^bpy^⋅⋅⋅Cl distances (3.595(4) Å and 3.605(4) Å) serve as a more reliable measure of the binding pattern. More importantly, the X‐ray crystal structure provides an experimental proof of the 1:1 HB bonding ability and, thus, enables the assessment of the results from theoretical calculations based on DFT (Supporting Information). Thus, the structural features of **5⋅Cl** were reproduced in very good agreement: the twist between the pyridine and triazole (8°), the C^bpy^ **⋅⋅⋅**Cl distance (3.407 Å), and the C‐H^trz^ **⋅⋅⋅**Cl angles (157.3°). More importantly, these marginal structural deviations confirm the suitability of DFT to assist the comprehensive discussion of the anion binding (vide infra).

### NMR titration

**Figure 3:**
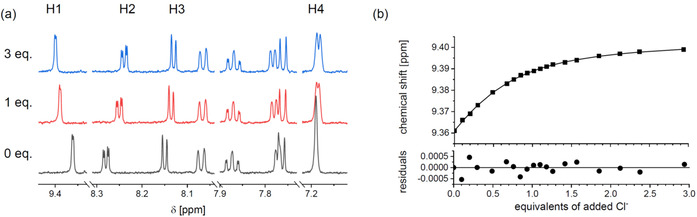
a) Fit results for the H1 peak (*K*_11_=7.0×10^4^ m ^−1^ and *K*_21_=1.0×10^4^ m ^−1^, 1:2 model). See Figure S31 of the Supporting Information for fit with 1:1 model. b) Aromatic region of selected ^1^H NMR spectra (600 MHz, CD_3_CN) during the titration of **6** with 0, 1, and 3 equiv of TBACl. Guest equivalents refer to the sensor concentration ([H]_0_=4.9×10^−5^ m). See Supporting Information for more details.

First, the scope of anion binding was tested by ^1^H NMR spectroscopy with XB‐based receptor **4** and the Ir‐based congener **6**. Standard NMR instrumentation enabled the titration of free sensor **4** with tetrabutylammonium bromide (TBABr) in CD_2_Cl_2_, which was selected to ensure sufficient solubility of the added guest in the desired concentration regime.^[30]^ The anticipated weak association (*K*_a_=15 m ^−1^) was obtained from fitting the H1 peak evolution with the BindFit program (Figure S11 of the Supporting Information). This value is reasonable, considering the lack of preorganization of the bpy subunit and the absence of cooperative charge assistance. Hence, the strongest binding is expected for the combination of the monocationic XB sensor **6** with the charge‐dense chloride anion. For reliable data interpretation both a sufficient signal‐to‐noise ratio and the diagnostic curvature regime in the binding curve are essential. As a consequence, advanced NMR instrumentation for the required dilute conditions becomes mandatory (see Supporting Information),^[3]^ which usually imposes instrumental challenges for routine NMR determination of high association. For this task, a 600 MHz spectrometer equipped with a cryoprobe enabled us to investigate binding constants in range up to 10^5^ m ^−1^ by accumulating only 128 scans per spectrum (a few minutes). Figure 3 shows selected NMR spectra and the diagnostic shifts of the H1 proton around 9.4 ppm. The observed downfield shift parallels that of the related bis‐*N*‐alkylated bpy‐based congener^[8b]^ or the pristine [Ir(bpy)(ppy)]^+^ complex.^[19]^ Notably, the curve fitting of H1 with BindFit^[31]^ required a 2:1 model to avoid larger and systematically distributed residuals (see Figure S12 of the Supporting Information for the 1:1 fit). The calculated association constants are *K*_11_=7.0×10^4^ m ^−1^ and *K*_21_=1.0×10^4^ m ^−1^. The *K*_11_ value is one order of magnitude larger than that for the structurally related carbazole‐based XB receptor (see Figure 1 a, *K*_11_=2.3×10^3^ m ^−1^),^[8d]^ which is assigned to the lack of charge assistance by the absent monocationic Ir fragment. Notably, similar polarization and preorganization of the triazole motifs by two internal hydrogen bonds (Figure 1 a, dashed OH groups) led also to a 2:1 host‐to‐guest species with surprisingly similar association constants (*K*_11_=7.1×10^4^ m ^−1^ and *K*_21_=3.2×10^3^ m ^−1^) in THF.^[8d]^ Furthermore, the anion binding capability of monocationic **6** even reaches that of related dicationic XB sensors, for example, the bis‐*N*‐alkylated analogues of Beer et al.^[8b]^ (Figure 1 b) and rotaxanes^[7b]^ (Figure 1 c). Importantly, the NMR titration confirms the anticipated XB behavior, and a more comprehensive discussion is provided below in conjunction with the corrected emission titration data.

### Optical titration

The anion recognition capability of HB sensor **5** and the related XB sensor **6** is presented for three typical anions: the charge‐dense chloride (Cl^−^) anion, the larger spherical bromide (Br^−^) anion, and the Y‐shaped oxygen‐based acetate (OAc^−^) anion. For this task, the corresponding cationic sensors with noncoordinating PF_6_^−^ counterions were titrated in the appropriate concentration regime (see Supporting Information Section 7). Since the absorption data changed only marginally, the emission data are evaluated in the following. Notably, it became evident during the course of this study that the emission data displayed an unusual behavior during titration, which is frequently found in the literature (vide infra),[^21d^, ^22^] but has not yet assessed in detail to the best of our knowledge. Hence, such informative emission data often remained unanalyzed.

### Background effects

**Figure 4:**
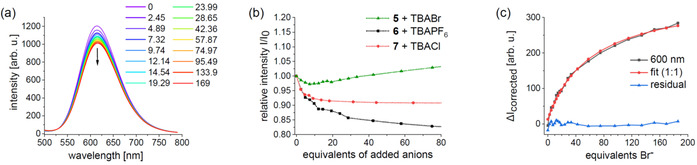
a) Raw emission data of the reference titration of **6** with noncoordinating TBAPF_6_. b) Titration profile (*λ*_em_=577 nm) of representative Ir complexes with selected anions. c) Corrected emission data of weak HB sensor **5** with the weak anion of TBABr including fit and residuals in a 1:1 model by using the BindFit program. Note the effect of correction of the raw data (green curve, b) and the corrected data (black curve, c) obtained for **5**+TBABr. Guest equivalents refer to the sensor concentration ([H]_0_=5×10^−6^ m for all experiments except for **6**+TBAOAc with [H]_0_=3×10^−6^ m). See Supporting Information for more details.

We began our systematic study by performing two control experiments. First, XB sensor **6** was titrated with noncoordinating TBAPF_6_ and the reference complex [Ir(bpy)(Meppy)_2_]^+^ (**7**) devoid of a binding motif with coordinating chloride. Figure 4 a shows representative 2D emission data for the reference experiments. For the sake of comparison, a wavelength (577 nm) was selected and scaled by its initial intensity to account for the different intrinsic quantum yields (Figure 4 b). Interestingly, XB sensor **6** shows a sizable emission decrease on addition of noncoordinating TBAPF_6_ (black curve, see also Figure S15 of the Supporting Information). A qualitatively similar behavior was found for reference complex **7** with the charge‐dense chloride anion, despite leveling at higher added equivalents (red curve, see also Figure S16 of the Supporting Information). The latter likely arises from an concomitant emission increase due to weak chloride binding, which has been reported for the related [Ir(bpy)(ppy)_2_]^+^ complex (*K*_a_=1.7×10^1^ m ^−1^).^[19]^ In line with that report, our titration experiments also confirmed conserved emission spectra of **6** and **7** irrespective of the anion. This finding is important to assign the emission changes on titration to the binding event rather than to changes in the excited‐state energetics. To omit speculation on the exact origin of such hitherto unknown quenching processes, we refer to this consistently observed effect as “background processes” in the following. For example, the titration of the weak HB sensor **5** with TBABr revealed an initial emission decrease on addition of approximately 7 equiv, followed by an overall increase in the later stage (Figure 4 b, green curve). Notably, attempting to fit such data leads to unmeaningful fits with the BindFit program.^[31]^ This general behavior is likely also the reason why reported spectral emission data are often presented without detailed analysis.[^21d^, ^22^] To exploit this otherwise very informative data, we used a simple correction procedure (see Supporting Information Section 7.3). In essence, each experimental emission data set was scaled according to the initial intensity of the reference titration (**6** with TBAPF_6_) before subtracting it, which also accounts for different intrinsic quantum yields of the Ir complexes. As a consequence, the corrected data (Δ*I*_corrected_) represent the spectral changes due to anion binding with respect to the pure sensor. Figure 4 c depicts the corrected data for a representative wavelength, including the obtained fit from a 1:1 model and the residuals to yield *K*_11_(**5⋅Br**)=3.2×10^3^ m ^−1^. Note that the quality of the fit is represented by small error values (0.4 %), which were obtained consistently for all subsequent fits and are significantly lower than those from the NMR titrations.

### HB and XB anion sensing

**Figure 5:**
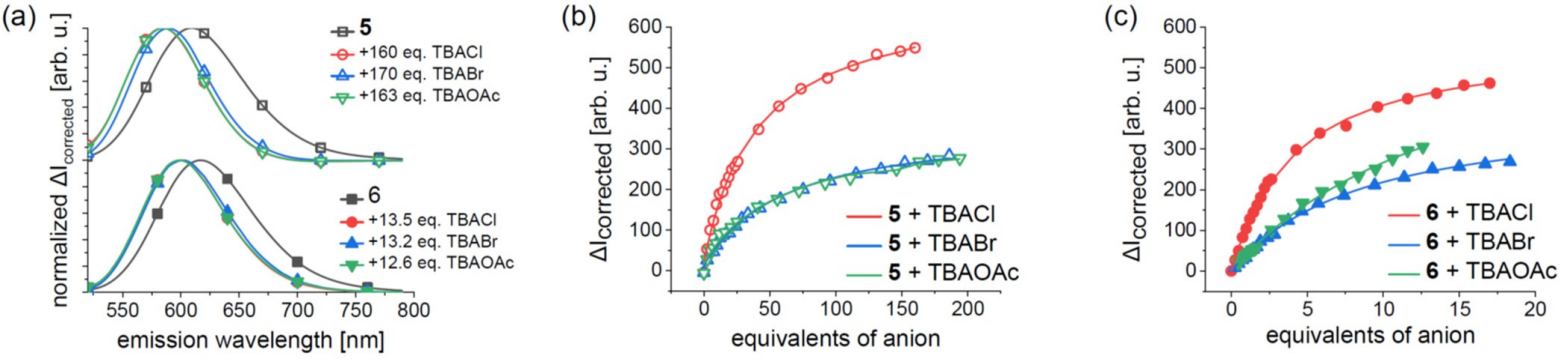
a) Emission spectra of HB sensor **5** (top, empty symbols) and XB sensor **6** (bottom, filled symbols), shown in black, and the shifted corrected emission spectra due to titration with TBACl (red circles), TBABr (blue triangle), and TBAOAc (green, triangle). Titration curves of b) HB sensor **5** and c) XB sensor **6** at 600 nm. Experimental data are shown as symbols and corresponding fits of the 1:1 model obtained with BindFit as lines. Guest equivalents refer to the sensor concentration ([H]_0_=5×10^−6^ m for all experiments except for **6**+TBAOAc with [H]_0_=3×10^−6^ m). See Supporting Information for more details.

Next, the remaining combinations of HB sensor **5** and XB sensor **6** with TBACl, TBABr, and TBAOAc were titrated, corrected by using the established correction procedure, and analyzed by a global fit from 550 to 700 nm with the BindFit program. Figure 5 a shows the diagnostic shifts of the corrected emission, which is more pronounced for HB sensor **5** (top) than XB sensor **6** (bottom). In general, the spectral emission shifts were comparable for chloride and acetate, whereas bromide led to somewhat smaller changes. This effect is tentatively assigned to the size of anion, and is discussed in more detail in the theoretical section (vide infra). The corresponding titration curves of both sensors are depicted for a single representative wavelength (Figure 5 b and c), including the corresponding fit results for a 1:1 model. The calculated *K*_a_ values are summarized in Table 1. For the HB sensor **5**, the obtained *K*_11_(**5⋅Cl**) value of 4.9×10^3^ m ^−1^ is more than one order of magnitude larger than that reported for [Ir(bpy)(ppy)_2_]^+^ (*K*_11_=1.7×10^2^ m ^−1^),^[19]^ which lacks the triazole motifs and only displays H^bpy^ **⋅⋅⋅**Cl interactions. The association of acetate is comparable [*K*_11_(**5⋅⋅OAc**)=3.2×10^3^ m ^−1^] to that of bromide and only slightly smaller than that for chloride, but stronger in comparison with [Ir(bpy)(ppy)_2_]^+^ with chloride. In the case of XB sensor **6**, the same trend was obeyed but the association constants were increased by one order of magnitude in comparison with the HB sensor. The largest value was reached for chloride [*K*_11_(**6⋅⋅Cl**)=5.7×10^4^ m ^−1^], which agrees reasonably well with that independently obtained from the NMR titration (7.0×10^4^ m ^−1^). Notably, the contribution of a 2:1 species is too small, because the concentrations in the emission experiments are one order of magnitude lower. More importantly, these *K*_a_ values are on the same order as the high ones reported for related rotaxanes and bis‐*N*‐alkylated congeners[^7b^, ^8b^] or a specific phosphate sensor (vide supra).[^22^, ^23^]

**Table 1:** Overview of the *K*_a_ values determined from ^1^H NMR data and emission data.

Host		Guest	Solvent	*K*_11_ [m ^−1^]	Titration method
**4**	XB	Br^−^	CD_2_Cl_2_	1.5×10^1^ (2.8 %)^[a]^	^1^H NMR
**6**	XB	Cl^−^	CD_3_CN	K_11_=7.0×10^4^ (5.2 %)^[a,b]^ K_21_=1.0×10^4^ (17.6 %)^[a,b]^	^1^H NMR
**5**	HB	Cl^−^	CH_3_CN	4.9×10^3^ (0.3 %)^[c]^	emission
	HB	Br^−^	CH_3_CN	3.2×10^3^ (0.4 %)^[c]^	emission
	HB	OAc^−^	CH_3_CN	3.2×10^3^ (0.6 %)^[c]^	emission
**6**	XB	Cl^−^	CH_3_CN	5.7×10^4^ (0.3 %)^[c]^	emission
	XB	Br^−^	CH_3_CN	2.5×10^4^ (0.3 %)^[c]^	emission
	XB	OAc^−^	CH_3_CN	2.2×10^4^ (0.2 %)^[c]^	emission

[a] By using the proton signals with *δ*>9.00 ppm. [b] Due to the increased host concentration on ^1^NMR titration, the 2:1 interaction can be identified, which is not found in the emission titration due to tenfold lower concentration. [c] With correction for the observed ionic effect (See Supporting Information). Fit: BindFit, UV 1:1 model.

### Limits of detection

The anion detection capability and the formal LODs were assessed (Table 2, see Supporting Information for more procedural details). The corrected emission data were interpolated to guest concentrations that would cause emission changes on the order of the (threefold) standard deviation of the measurement conditions.^[32]^ In this case, HB receptor **5** showed micromolar sensitivity, and XB receptor **6** exhibited excellent nanomolar LOD values (11–30 nm) for all three anions. These values are comparable to the best values reported by Ghosh et al. for specific phosphate sensing.^[22]^ As mentioned above, the LOD depends both on the recognition and the response, and thus on the sensitivity of the spectrometer, data accumulation, and so on. Hence, a practical estimate for fast data acquisition (1 s accumulation time at 600 nm) was derived by applying the standard deviation at 600 nm of the calibration curves to determine the real sensing capability (Table 2). Consequently, the values for the applied sensing capability and likewise the LODs can be significantly enhanced by prolonged acquisition time or, vice versa, anion detection can be simply achieved in much shorter time than performing ITC measurements. Notably, distinguishing the background effect from the recognition event is crucial and relies on the diagnostic wavelength shifts of the latter. To the best of our knowledge, this beneficial combination is of striking difference in comparison to more sophisticated methods, for example, NMR or ITC measurements.

**Table 2:** Anion sensing capability and LODs.

Host		Guest	Solvent	Host conc. [m]	Sensing capability^[a]^ [μm]	LOD^[b]^ [μm]
**5**	HB	Cl^−^	CH_3_CN	5.2×10^−16^	3.05	0.99
	HB	Br^−^	CH_3_CN	5.1×10^−6^	5.85	4.80
	HB	OAc^−^	CH_3_CN	5.3×10^−6^	7.90	1.95
**6**	XB	Cl^−^	CH_3_CN	5.1×10^−6^	0.25	0.011
	XB	Br^−^	CH_3_CN	5.1×10^−6^	0.50	0.030
	XB	OAc^−^	CH_3_CN	3.1×10^−6^	0.36	0.027

[a] Practical limit determined from the standard deviation (SD) of residual of the applied fitting scheme. [b] LOD as calculated from the threefold SD of a blank measurement. See Supporting Information Section 8.6 for details of calculations.

### Theoretical calculations

To comprehensively rationalize the anion binding capability and the observed emission changes, DFT calculations were performed. The suitability of this approach to reproduce the experimental X‐ray structure is presented for **5⋅Cl** (vide supra). For both sensor complexes **5** and **6**, the singlet ground state (^1^GS) and triplet metal‐to‐ligand‐charge‐transfer (^3^MLCT) state were computed, including those in the presence of chloride. In general, the structural features of the receptor‐decorated bpy subunit are discussed in terms of binding capability,^[33]^ and the emission characteristics are assessed in terms of electronic/energetic changes in the corresponding triplet states. A more detailed analysis and tabulated data are provided in the Supporting Information, and the main results are presented in the following. First, the HB sensor **5** has a negligible dihedral twist of the triazole and pyridine units (<1°), which increases to 8° on chloride binding. The related XB sensor **6** has a larger initial dihedral twist (16°) due to the larger iodine donor atoms, and likewise experiences a further increase (24 and 27°) to accommodate the chloride anion. The corresponding H**⋅⋅⋅**Cl and I**⋅⋅⋅**Cl distances are 2.378 and 2.980 Å, respectively. The C−H**⋅⋅⋅**Cl bond angle is 157°, and the C−I**⋅⋅⋅**Cl angle of 178° reproduces correctly the preference for linearity of XBs. The contribution of the bpy fragment (H^bpy^) to the anion binding differs among the two sensors; for **5⋅Cl** hydrogen bonding is observed, as judged from the coplanar arrangement and the short H^bpy^ **⋅⋅⋅**Cl distance (2.508 Å). In the case of the XB system, the dihedral twist of the binding motif leads to a strong out‐of‐plane displacement of the H^bpy^ and Cl atoms, that is, a spatial separation of 4.904 Å for **6⋅Cl**. Likewise, the charge assistance is higher for **5⋅Cl** than for **6⋅Cl**, as shown by the short distances between the Ir and Cl atoms of 7.407 and 9.129 Å, respectively. These findings confirm that the HB sensor **5** experiences additional HB contributions from the bpy fragment, in line with the reported crystal‐structure data of the [Ir(bpy)(ppy)_2_]^+^ fragment.^[19]^ Notably, this contribution is not possible for **6** due to the steric congestion. In the corresponding excited states, no major structural changes were observed, whereas the electronic structure revealed distinct differences with respect to the ground states and on anion binding.

**Figure 6:**
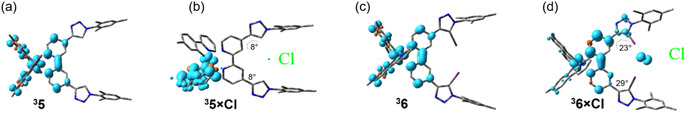
Spin density plots (isovalue drawn at 0.004) of the ^3^MLCT excited states of a) the HB receptor ^3^ **5** and b) in the presence of chloride ^3^ **5⋅Cl**, c) XB receptor ^3^ **6** and d) in the presence of chloride ^3^ **6⋅Cl**. Note the differences in localization of the unpaired electrons (blue areas).

The excited states can be well visualized by spin‐density plots, which show similar spin localization for ^3^ **5** (Figure 6 a) and ^3^ **6** (Figure 6 c) over the entire Ir fragment, which represents a formal charge transfer from electron‐rich Ir(ppy)‐based HOMO. In the case of ^3^ **5⋅Cl**, the anionic nature of chloride destabilizes the nearby LUMO and, thus, raises its energy. This is in line with the different spin localization, as depicted in Figure 6 b, which showed no bpy contribution. In the case of ^**3**^ **6⋅Cl**, the significantly longer distance of the chloride anion to the Ir(bpy) fragment leads to a less severe destabilization, and concomitantly increases the electron density at the triazole moiety. The latter results in an additional contribution to the HOMO at the cost of the ppy contribution. Nevertheless, a sizable raise in LUMO energy is still expected. Because the calculation of phosphorescence spectra is not trivial, the energies of the excited states with respect to the ground state are often evaluated (ΔSCF approach) as a versatile tool to identify systematic effects in qualitative fashion. The formal emission energies for the HB sensor **5** and XB sensor **6** are 2.19 eV and 2.16 eV, respectively. Although the difference is small, it reproduces the experimentally observed blueshifted emission (i.e., higher energy) of **5** compared with **6**. On anion binding, the formal emission energies of **5⋅Cl** and **6⋅Cl** are raised to 2.49 and 2.42 eV, respectively. Although this energy is overestimated due to the additional anion, it again reproduces the observed larger blueshift for the HB system (+0.30 eV) compared with the XB system (+0.25 eV). In summary, the DFT calculations reproduced the geometric and electronic features in a consistent manner, including the energetic changes in emission energy. Consequently, they are believed to serve as an indispensable tool to assist the analysis of emission data and, moreover, to fuel the design of future XB receptors for application in, for example, supramolecular chemistry, anion sensing, and catalysis.[^10a^, ^14b^, ^16a^] In this regard, the shifts in spectral emission wavelength add a new dimension to the analysis (in addition to mere intensity changes) and, thus, point towards the possibility of further discriminating the size and geometry of the anion, which is under current investigation. Finally, this new methodology is believed to be universal beyond Ir‐based emitters, and offers applications in photonic nanostructures for state‐of‐the art optical anion sensors.^[34]^

## Conclusion

A set of charge‐assisted luminescent anion sensors featuring HB or XB motifs was prepared. For the receptor part, a short and modular synthesis route was utilized to form the triazole under classical (HB system) or direct iodinating conditions (XB system) as the key step, followed by coordination of a monocationic Ir fragment as the luminophore in overall high yields. The X‐ray crystallographic analysis of the HB receptor with bound chloride confirmed the anticipated bidentate coordination mode for anions. Applying advanced cryoprobe NMR instrumentation reduced the measurement time to 5 min per data point under the required dilute conditions. The analysis revealed very large *K*_a_ values (10^4^ to 10^5^ m ^−1^) for the XB of chloride, in excellent agreement with reported values of related receptors.

As the main result, the scope of emission detection of the HB and XB receptors was elucidated. First, the contribution of unspecific quenching was quantified with noncoordinating PF_6_^−^. The preserved emission maxima enables the reliable correction of the emission changes behavior in case of specific anion binding, as exemplified in detail for chloride, bromide, and acetate. Second, true anion binding led to diagnostic blueshifted emission and increased quantum yield, which was more pronounced for the HB system compared to the XB system. As consequence, excellent fits using the available BindFit program could be obtained, which revealed *K*_a_ values for the HB system in the range of 10^3^ to 10^4^ m ^−1^, and for the XB system of 10^4^ to 10^5^ m ^−1^_._ For both receptors, chloride was superior to bromide and acetate. Notably, the validity of the emission correction procedure is demonstrated by the consistent values obtained from NMR titration. Third, the spectral emission shift could be consistently corroborated by DFT calculations. The computational results further showed that the HB receptor can adopt an almost ideal planar receptor geometry for chloride, which is ideal to maximize the electronic perturbation of the emissive part on binding. For the XB system, a sizable out‐of‐plane deformation of the emissive and the receptive subunits was found, which explained the less pronounced blueshifted emission. These consistent findings underpin the power of a DFT‐assisted design approach of the binding cavities and the luminophore, mediated by their electronic communication.

For the first time, a consistent emission‐based analysis for bidentate HB and XB anion receptors has been presented. Such an emission‐based methodology is highly attractive to determine large *K*_a_ values, which are typically inaccessible for NMR spectroscopy or require sophisticated instrumentation (e.g., ITC). Notably, this simple, fast, and widely available instrumental technique is ideally suited after background correction for analysis by open‐source software (e.g., BindFit). Future directions may include extending the set of guests to biologically relevant anions, for example, phosphate‐based anions, as well as to decrease further the specific (nanomolar) limit of detection, which is tuned by the precise mutual interaction of the binding event and the emissive part.

## Experimental Section

For experimental details concerning the synthesis, analytical characterization, NMR spectra, DFT calculations, titration data from NMR and emission spectroscopy, and X‐ray crystallographic data, see the Supporting Information. Deposition Number(s) 1962576 and 1962577 contain the supplementary crystallographic data for this paper. These data are provided free of charge by the joint Cambridge Crystallographic Data Centre and Fachinformationszentrum Karlsruhe Access Structures service www.ccdc.cam.ac.uk/structures.

## Conflict of interest

The authors declare no conflict of interest.

## Supporting information

As a service to our authors and readers, this journal provides supporting information supplied by the authors. Such materials are peer reviewed and may be re‐organized for online delivery, but are not copy‐edited or typeset. Technical support issues arising from supporting information (other than missing files) should be addressed to the authors.

Supplementary
