# Supplementary material for: Facile and Reliable Emission‐Based Nanomolar Anion Sensing by Luminescent Iridium Receptors Featuring Chelating Halogen‐Bonding Sites
Source: Chemistry. 2020 Oct 7;26(64):14679–87. doi: 10.1002/chem.202002738 (PMC7756348; doi:10.1002/chem.202002738)
Supplement: Supplementary file 1 — Supplementary [file CHEM-26-14679-s001.pdf]

Chemistry–A European Journal

Supporting Information

Facile and Reliable Emission-Based Nanomolar Anion Sensing by Luminescent Iridium Receptors Featuring Chelating Halogen-Bonding Sites

Robin Kampes,[a, b] Ronny Tepper,[a, b, e] Helmar Görls,[c] Peter Bellstedt,[a, c] Michael Jäger,[a, d] and Ulrich S. Schubert*[a, b, d]

Content

1. Instrumentation 2
2. Materials 2
3. Synthesis 2
4. NMR spectra 5
5. X-ray crystallography 9
6. DFT calculations 10
7. NMR data 12
7.1. NMR titration of free XB sensor 4 with TBABr 13
7.2. NMR titration of 6 13
7.3. NMR evaluation of XB sensor 6 with TBACl using a 1:1 model 14
8. Optical data 14
8.1. Absorption and emission spectra 16
8.2. Reference titration experiments 16
8.3. Emission correction procedure 17
8.4. Emission titration of HB sensor 5 18
8.5. Emission titration of XB Sensor 6 21
8.6. Limit of detection 23
9. References 25

1. Instrumentation

NMR spectra were recorded on Bruker spectrometer (300, 400, 500, and 600 MHz) in deuterated solvents (Eurisotop) at 298.1 K. ESI MS spectra were measured using a Bruker ESI-(Q)-TOF microTOF II mass spectrometer. The mass spectrometer was operating in the positive ion mode and the standard electrospray ionization (ESI) source was used to generate the ions. UV/vis measurements were carried out at a Perkin Elmer Lambda45 instrument, using quartz glass cuvettes (d = 10 mm).

2. Materials

4,4'-*Bis*((triisopropylsilyl)ethynyl)-2,2'-bipyridine (**1**)[1], mesityl azide (**2**)[2] and $[Ir(Meppy)_2Cl]_2$ (Meppy is 2-(2-methyl-phenyl)pyridine)[3] were synthesized according to literature procedures. All further chemicals were purchased from Sigma Aldrich, Alpha Aesar, ABCR and TCI, and used as delivered. Marked compounds were purified using a Biotage Isolera One flash chromatography system with Biotage SNAP KP-Sil cartridges. TLC was performed with silica gel on alumina sheets 20 × 20 cm from Merck. Dry solvents were bought in sealed bottles from Acros Organics and Sigma Aldrich. For the titration experiments *tetra*-*N*-butylammonium chloride (99%), bromide (99%) and acetate (97%) were purchased in high purity and previously dried in a desiccator (over phosphorus pentoxide), while Acros Organics acetonitrile 99.9% extra dry was used as solvent.

3. Synthesis

4,4'-*Bis*(1-mesityl-1H-1,2,3-triazol-4-yl)-2,2'-bipyridine (3)

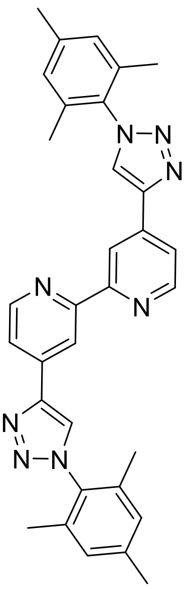

A mixture of CH_2Cl_2 (8 mL), water (8 mL) and ethanol (16 mL) was purged with nitrogen for 15 min. Successively, the azide **2** (850 mg, 5.3 mmol, 2.4 eq.), sodium ascorbate (869 mg, 4.4 mmol, 2 eq.), copper(II) sulfate (70 mg, 0.4 mmol, 0.2 eq.) as well as **1** (448 mg, 2.2 mmol, 1 eq.) were added to the solution. Under exclusion of light the solution was stirred for 3.5 h at 50 °C and subsequently 20 h at 35 °C. After addition of more sodium ascorbate (435 mg, 2.2 mmol, 1 eq.) as well as copper(II) sulfate (35 mg, 0.2 mmol, 0.1 eq.) the reaction mixture was stirred for further 3 h at 50 °C. 20 drops of *N*-(2-hydroxyethyl)ethylenediaminetriacetic acid (HEEDTA) were added and after cooling the reaction mixture was poured into water (35 mL) and extracted with CH_2Cl_2. The organic layer was dried over Na_2SO_4 and the solvent was evaporated under reduced pressure. Afterwards, a white and a black fraction of product were obtained by gel filtration (silica, CH_2Cl_2 with 5% MeOH). The black fraction was dissolved in CH_2Cl_2 and washed with aqueous HEEDTA

solution to obtain white powder. Both fractions were combined to yield the title compound (1.077 g, 2.1 mmol, 93%).

^{1}H NMR (300 MHz, $CDCl_3$): δ = 8.84 – 8.76 (m, 4H), 8.17 (s, 2H), 8.10 (dd, J = 5.1, 1.6 Hz, 2H), 7.04 (s, 4H), 2.39 (s, 6H), 2.03 (s, 12H) ppm.

^{13}C NMR (75 MHz, $CDCl_3$): δ = 156.4, 150.0, 145.3, 140.4, 139.1, 135.0, 133.2, 129.2, 123.6, 120.2, 117.6, 21.2, 17.4 ppm.

HRMS (ESI-TOF) m/z: $[M+H]^+$ calcd. for $C_{32}H_{30}N_8$, 527.2666; found, 527.2641. Error: 4.9 ppm.

4,4'-*Bis*(5-iodo-1-mesityl-1H-1,2,3-triazol-4-yl)-2,2'-bipyridine (4)

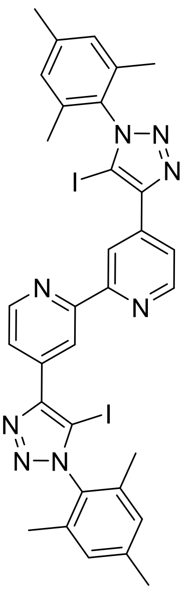

A two-necked round bottom flask containing **2** (124 mg, 0.8 mmol, 2.25 eq.) was purged with nitrogen and wrapped with aluminum foil to exclude light. In succession THF (2.5 mL), sodium iodide (411 mg, 2.7 mmol, 8 eq.), copper(II) perchlorate (508 mg, 1.4 mmol, 4 eq.), *tris*((1-benzyl-4-triazolyl)-methyl)amine (36 mg; 0.07 mmol, 0.2 eq.), 1,8-diazabicyclo[5.4.0]undec-7-ene (104 mg, 0.7 mmol, 2 eq.) dissolved in THF (1 mL), and **1** (70 mg, 0.3 mmol, 1 eq.) were added to the reaction mixture. After 17 h stirring at room temperature full conversion was confirmed with TLC whereon the reaction mixture was poured into CH_2Cl_2, washed twice with aqueous NaOH (25%) and brine each, dried over Na_2SO_4 and the solvent was evaporated under reduced pressure. Unreacted azide was separated from the crude product with gel filtration (silica, 1st CH_2Cl_2, 2nd CH_2Cl_2 with 5% MeOH). The pure product (215 mg, 0.28 mmol, 81%) was obtained after twofold column chromatography using the chromatography system (1st: silica, CH_2Cl_2 with 0 to 5% MeOH no separation; 2nd: CH_2Cl_2 with 30 to 100% ethyl acetate).

^{1}H NMR (500 MHz, CD_2Cl_2): δ = 9.29 (dd, J = 1.8, 0.8 Hz, 2H), 8.84 (dd, J = 5.1, 0.9 Hz, 2H), 8.12 (dd, J = 5.1, 1.8 Hz, 2H), 7.11 (s, 4H), 2.42 (s, 6H), 1.96 (s, 12H) ppm.

^{13}C NMR (126 MHz, CD_2Cl_2): δ = 157.0, 150.4, 147.7, 141.7, 139.4, 136.5, 133.3, 129.8, 121.8, 118.8, 81.8, 21.6, 17.8 ppm.

HRMS (ESI-TOF) m/z: $[M+H]^+$ calcd. for $C_{32}H_{28}I_2N_8$, 779.0599; found, 779.0609. Error: -1.2 ppm.

General procedure for Ir sensors (5 and 6)

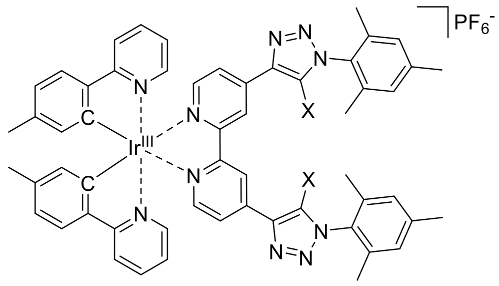

The sensors **5** and **6** were synthesized analog to literature procedure.[4] 0.5 eq. of precursor [Ir(Meppy)$_2$Cl]$_2$ and 1 eq. ligand **3** or **4** were dissolved in a mixture of CH_2Cl_2 (8 mL) and methanol (10 mL). The reaction mixture was stirred at 50 °C for 4 h. After cooling to room temperature, saturated aqueous NH_4PF_6 solution (2 mL) was added and the reaction mixture was stirred overnight. Subsequently, the reaction mixture was evaporated, resolved in CH_2Cl_2, washed three times with water, dried over Na_2SO_4 and precipitated from pentane. The product was obtained from gel filtration (aluminum oxide, CH_2Cl_2 with 2.5% acetone).

Table S1. Details of the synthesis of Ir complexes 5 (X = H) and 6 (X = I).

X	Precursor	Ligand	Yield
H	38 mg, 0.03 mmol	**3**, 35 mg, 0.07 mmol	68 mg, 0.06 mmol, 85%
I	33 mg, 0.03 mmol	**4**, 45 mg, 0.06 mmol	71 mg, 0.05 mmol, 85%

Sensor 5 (X = H):

^{1}H NMR (500 MHz, $CDCl_3$): δ = 8.97 (s, 2H), 8.86 (s, 2H), 8.29 (d, J = 5.8 Hz, 2H), 7.99 (d, J = 5.8 Hz, 2H), 7.90 (d, J = 8.2 Hz, 2H), 7.75 (t, J = 7.8 Hz, 2H), 7.63 (d, J = 7.9 Hz, 2H), 7.59 (d, J = 5.9 Hz, 2H), 7.00 – 6.93 (m, 6H), 6.90 (d, J = 8.0 Hz, 2H), 6.14 (s, 2H), 2.32 (s, 6H), 2.18 (s, 6H), 1.98 (s, 12H) ppm.

^{13}C NMR (126 MHz, $CDCl_3$): δ = 168.2, 156.3, 150.9, 150.7, 148.4, 142.9, 142.2, 141.3, 140.9, 140.3, 138.0, 135.1, 133.1, 132.7, 129.2, 127.3, 124.9, 124.0, 124.0, 122.8, 121.4, 119.4, 77.2, 22.1, 21.3, 17.3 ppm.

HRMS (ESI-TOF) m/z: $[M]^+$ calcd. for $C_{56}H_{50}IrN_{10}$, 1055.3844; found, 1055.3813. Error: 3.5 ppm.

Sensor 6 (X = I):

^{1}H NMR (400 MHz, CD_2Cl_2): δ = 9.39 (d, J = 1.9 Hz, 2H), 8.33 (dd, J = 5.8, 1.7 Hz, 2H), 8.16 (d, J = 5.8 Hz, 2H), 7.96 (d, J = 8.1 Hz, 2H), 7.80 (td, J = 7.9, 1.5 Hz, 2H), 7.69 (d, J = 8.0 Hz, 2H), 7.62 (dd, J = 5.9, 1.4 Hz, 2H), 7.10 (s, 4H), 7.04 – 6.96 (m, 2H), 6.95 (dd, J = 8.0, 1.6 Hz, 2H), 6.18 (s, 2H), 2.41 (s, 6H), 2.18 (s, 6H), 1.92 (d, J = 1.9 Hz, 2H) ppm.

^{13}C NMR (101 MHz, CD_2Cl_2): δ = 168.4, 156.5, 151.7, 150.7, 148.9, 145.0, 142.1, 141.9, 141.8, 141.5, 138.7, 136.2, 136.2, 132.9, 132.9, 129.9, 125.5, 125.1, 124.4, 123.3, 121.4, 120.2, 22.1, 21.6, 17.7 ppm.

HRMS (ESI-TOF) m/z: $[M]^+$ calcd. for $C_{56}H_{48}I_2IrN_{10}$, 1307.1777; found, 1307.1732. Error: 3.7 ppm.

$[Ir(bpy)(Meppy)_2]PF_6$ (7)

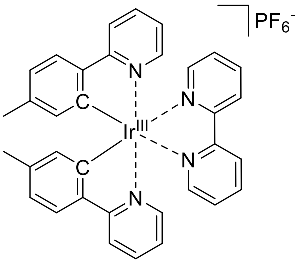

Reference complex **7** was synthesized analog to **5** or **6** except precipitation in pentane.

^{1}H NMR (400 MHz, CD_2Cl_2): δ = 8.49 (d, J = 8.1 Hz, 2H), 8.12 (td, J = 7.9, 1.6 Hz, 2H), 8.03 (dd, J = 5.6, 1.7 Hz, 2H), 7.90 (d, J = 8.2 Hz, 2H), 7.75 (td, J = 7.8, 1.5 Hz, 2H), 7.64 (d, J = 7.9 Hz, 2H), 7.50 – 7.43 (m, 4H), 7.01 – 6.86 (m, 4H), 6.11 (s, 2H), 2.15 (s, 6H) ppm.

^{13}C NMR (101 MHz, CD_2Cl_2): δ = 168.4, 156.2, 151.3, 150.7, 148.8, 141.8, 141.5, 139.8, 138.6, 132.9, 128.8, 125.4, 125.0, 124.3, 123.2, 120.0, 22.1 ppm.

HRMS (ESI-TOF) m/z: $[M]^+$ calcd. for $C_{34}H_{28}IrN_4$, 685.1938; found, 685.1949. Error: 1.2 ppm.

4. NMR spectra

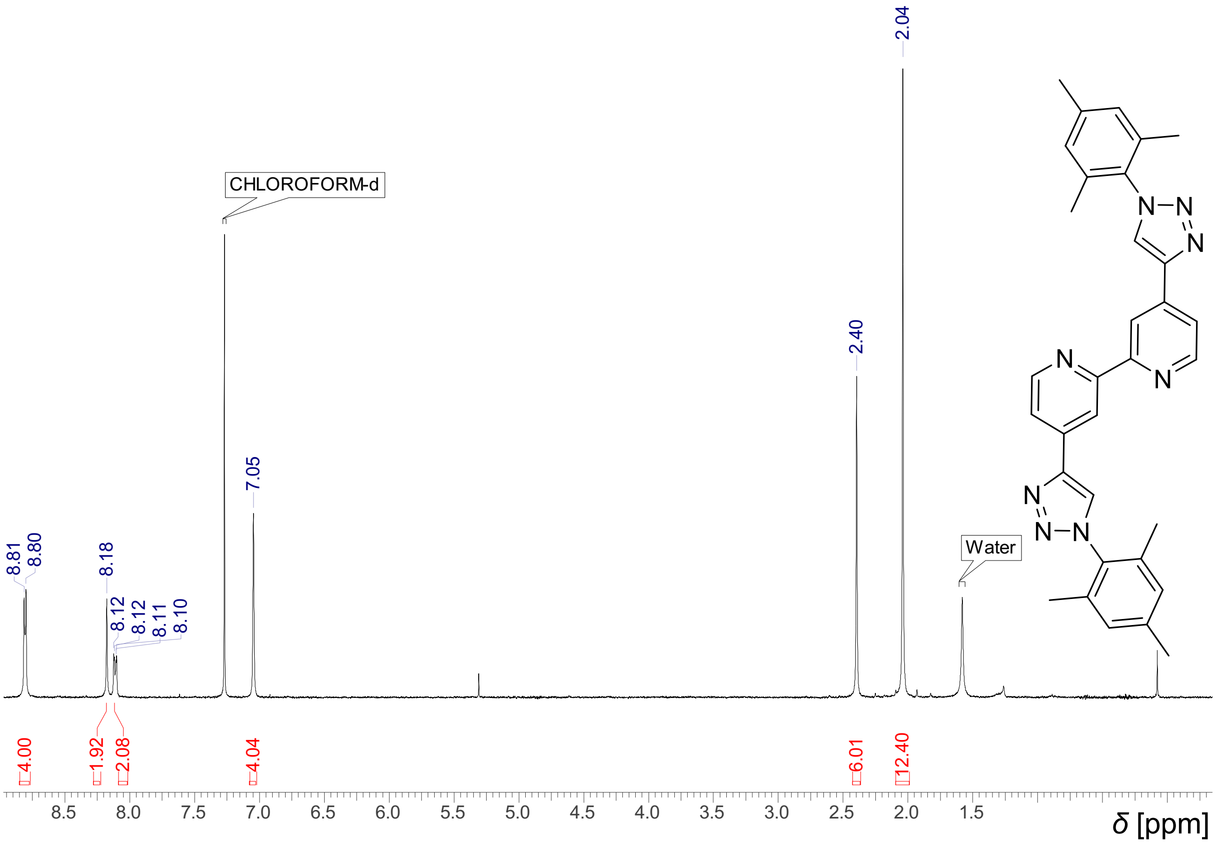

*Figure S1. ^{1}H NMR spectrum of compound **3** (300 MHz, $CDCl_3$).*

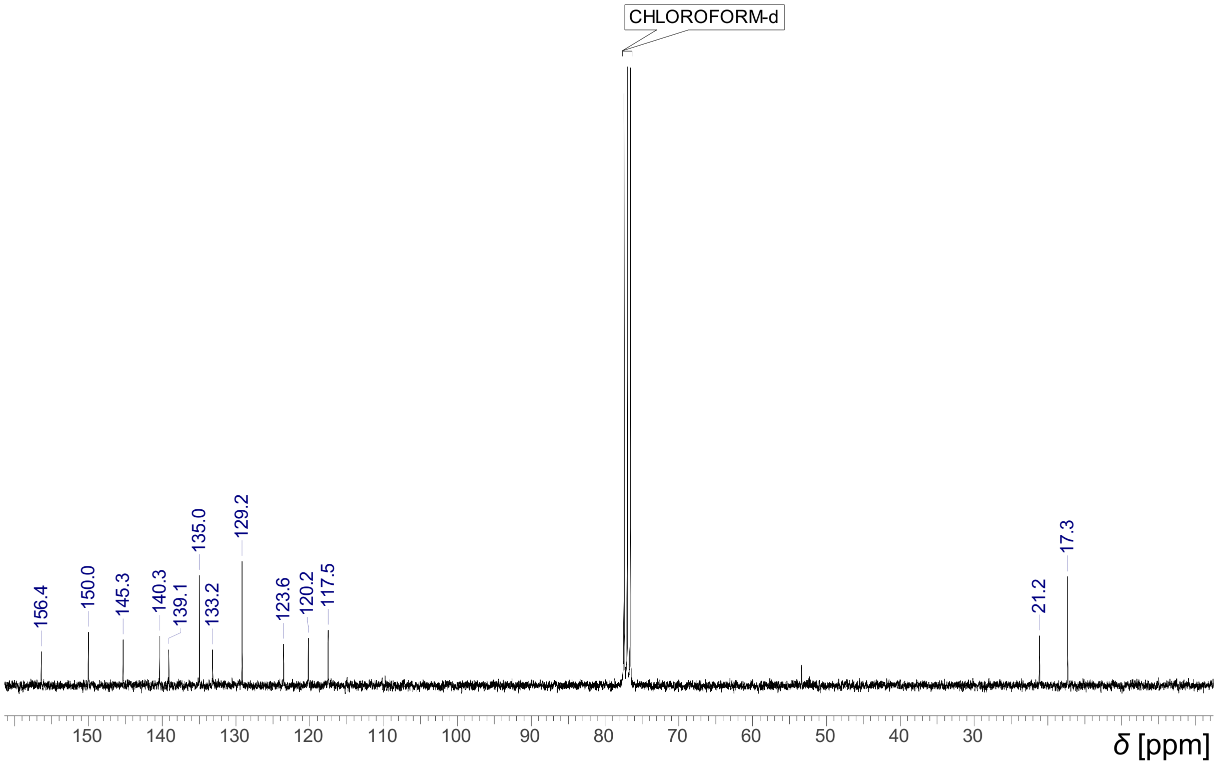

*Figure S2. ^{13}C NMR spectrum of compound **3** (75 MHz, $CDCl_3$).*

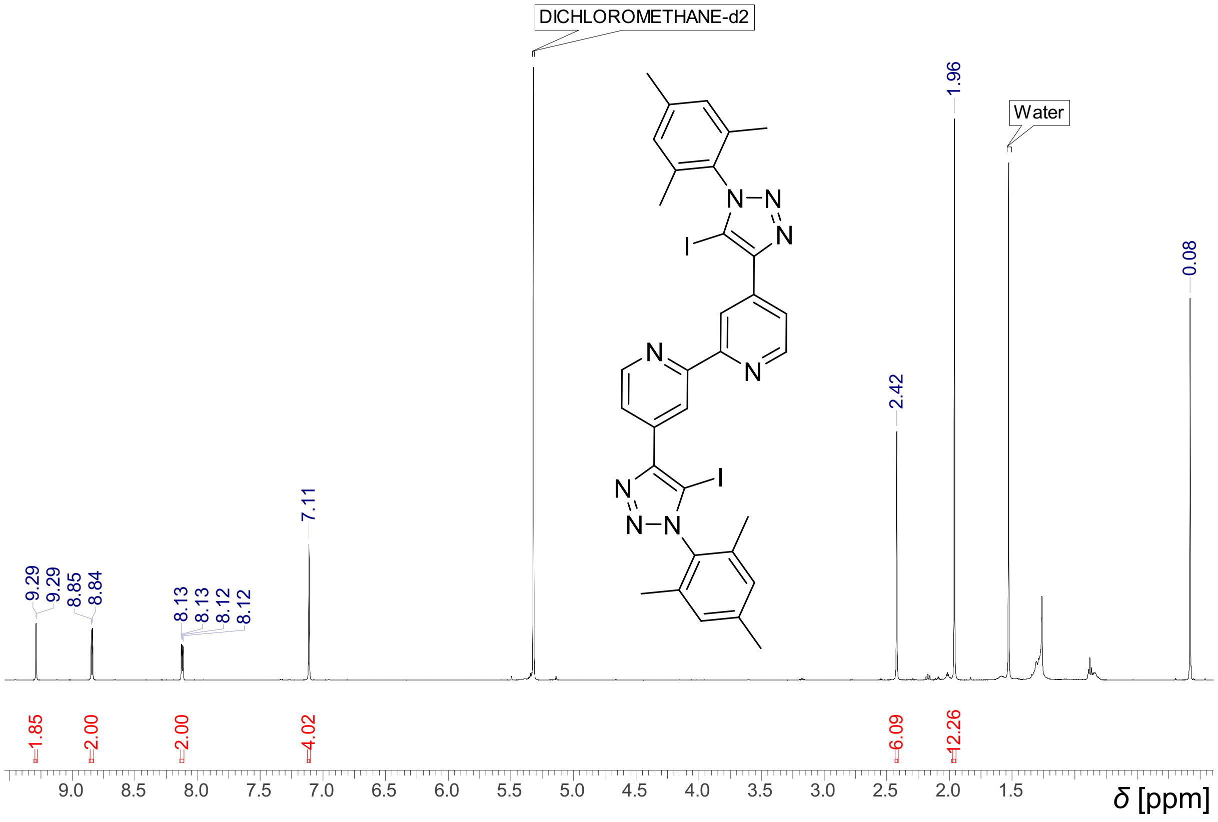

*Figure S3. ^{1}H NMR spectrum of compound **4** (500 MHz, CD_2Cl_2).*

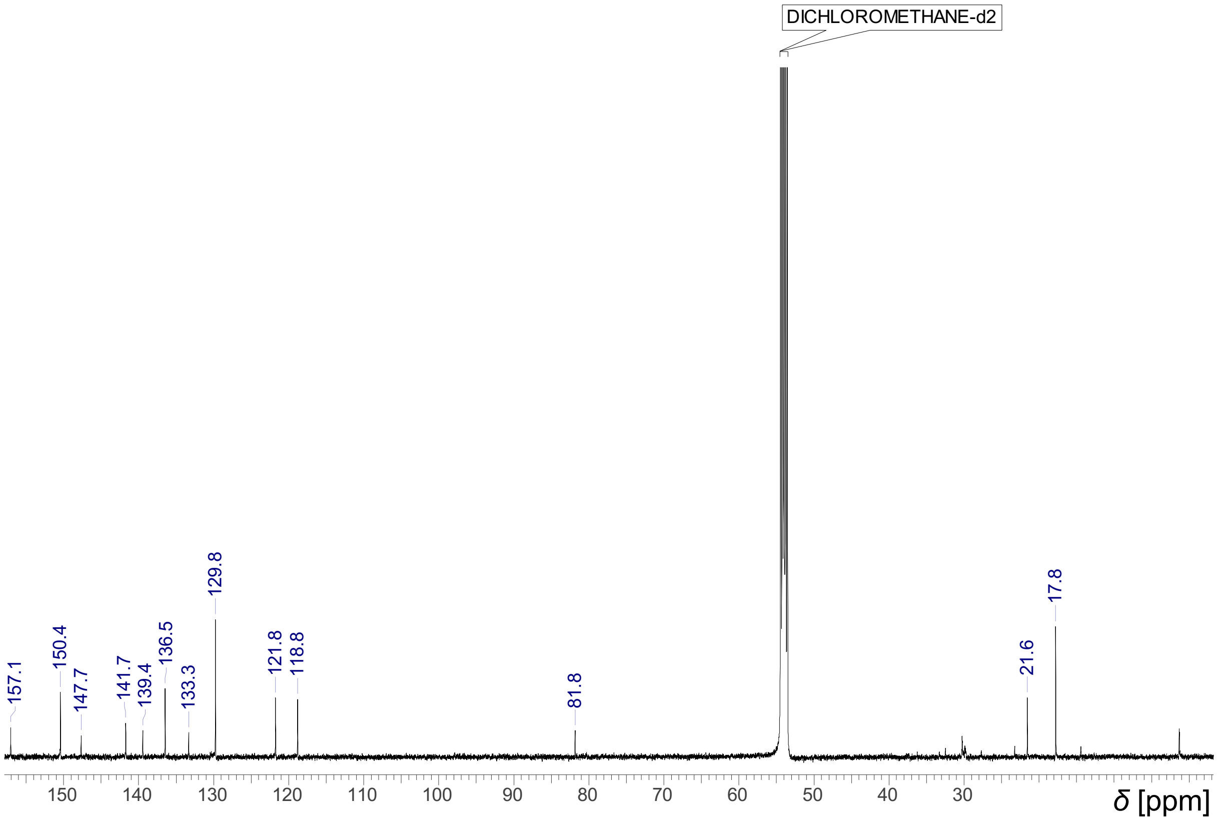

*Figure S4. ^{13}C NMR spectrum of compound **4** (126 MHz, CD_2Cl_2).*

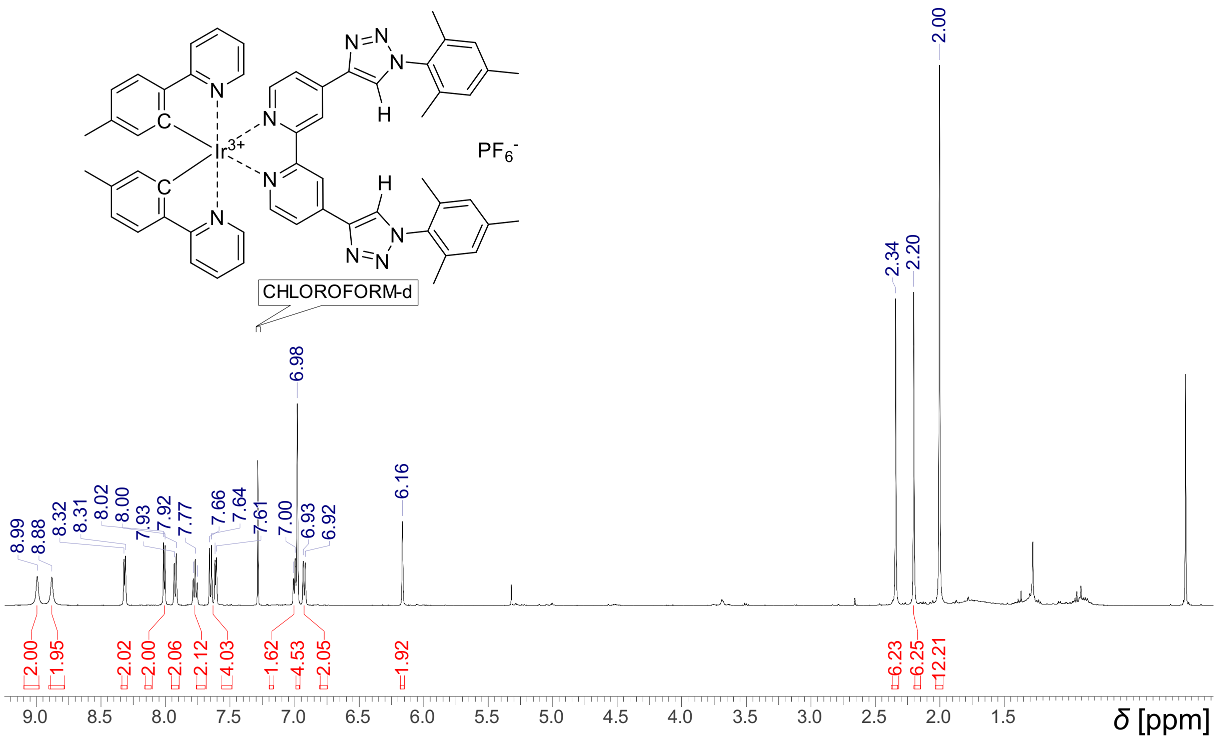

*Figure S5. ^{1}H NMR spectrum of compound **5** (500 MHz, $CDCl_3$).*

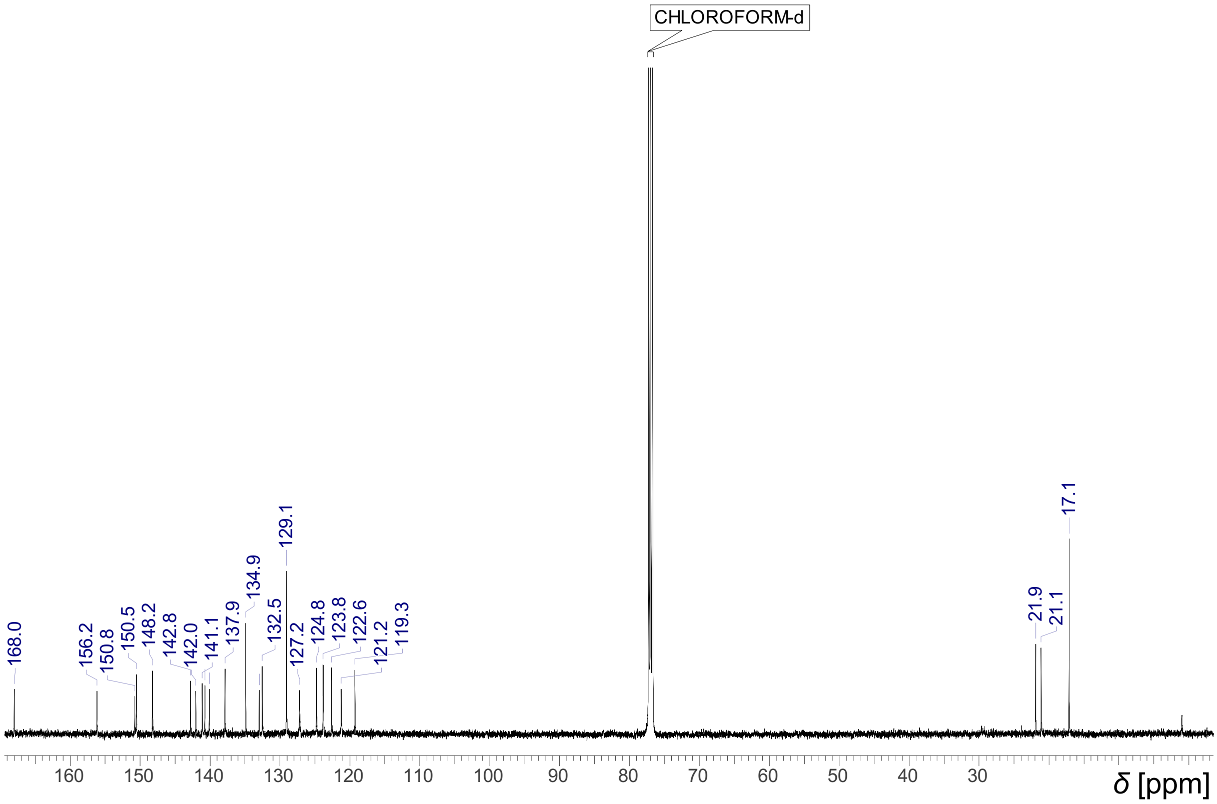

*Figure S6. ^{13}C NMR spectrum of compound **5** (126 MHz, $CDCl_3$).*

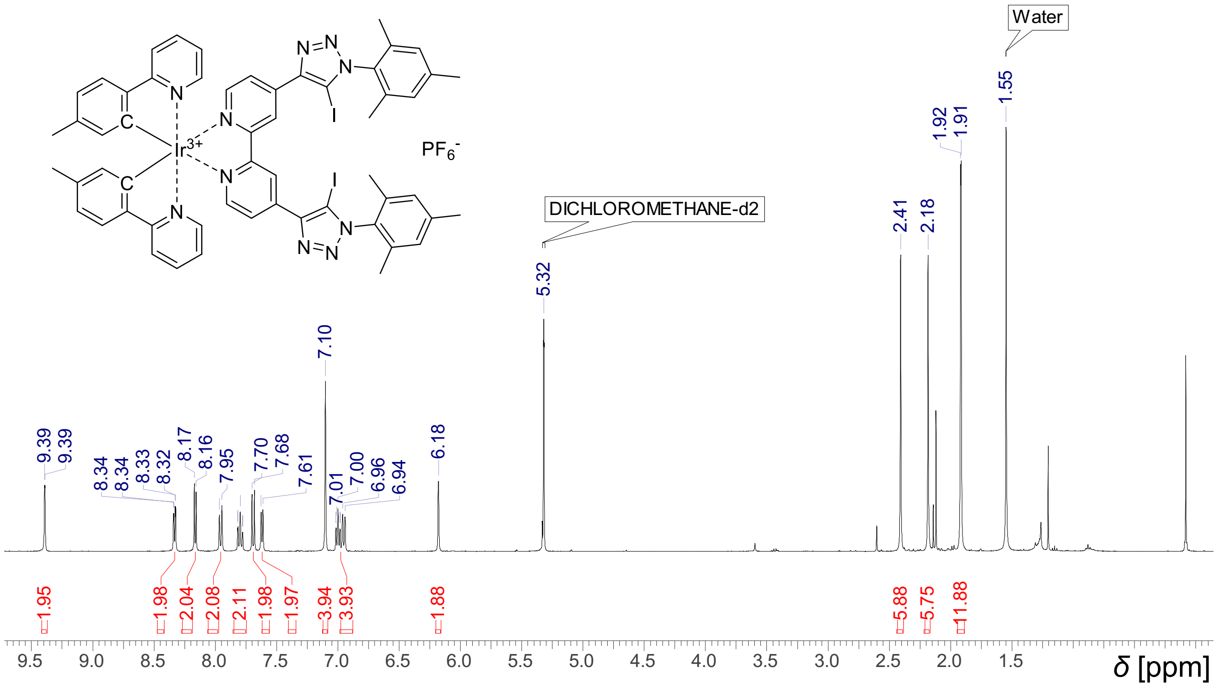

*Figure S7. ^{1}H NMR spectrum of compound **6** (400 MHz, CD_2Cl_2).*

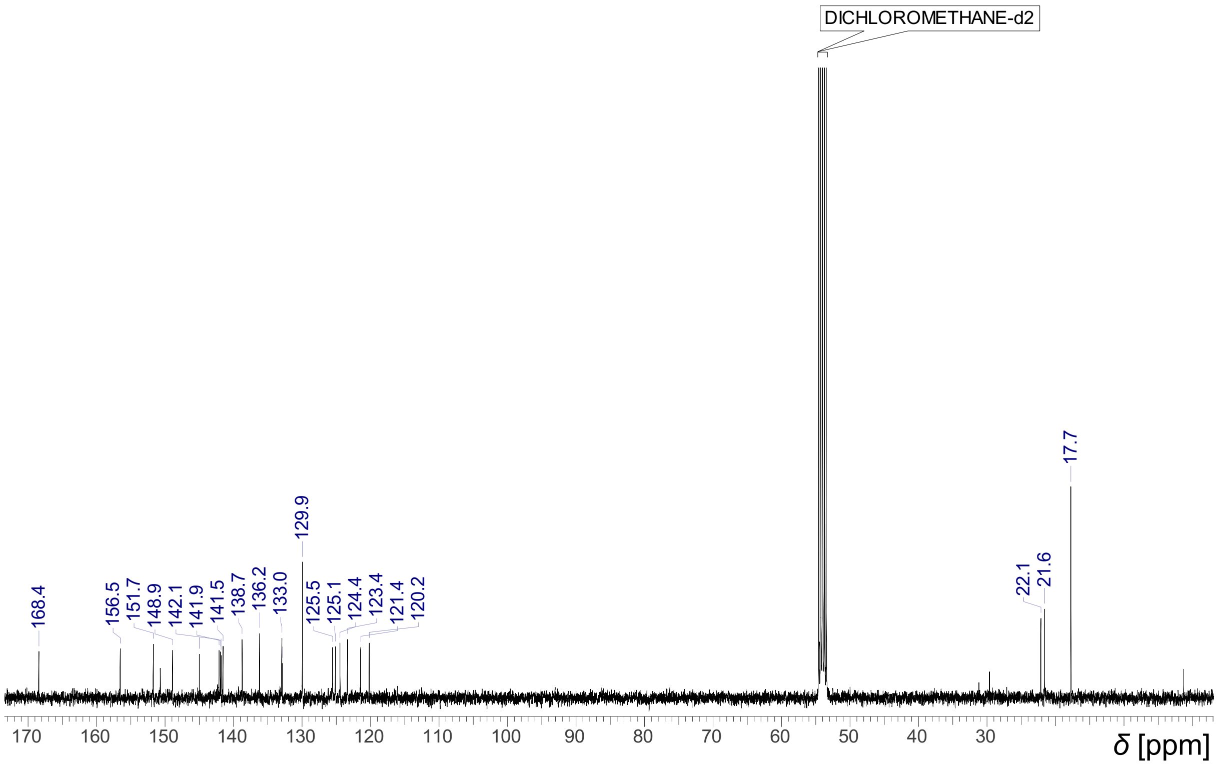

*Figure S8. ^{13}C NMR spectrum of compound **6** (101 MHz, CD_2Cl_2).*

5. X-ray crystallography

Crystal Structure Determination. The intensity data were collected on a Nonius KappaCCD diffractometer, using graphite-monochromated Mo-K_α radiation. Data were corrected for Lorentz and polarization effects; absorption was taken into account on a semi-empirical basis using multiple-scans.[5-7] The structure was solved by direct methods (SHELXS[8]) and refined by full-matrix least squares techniques against Fo^2 (SHELXL-97[8]). The hydrogen atom bonded to triazole carbon atom C7 of **3** was located by difference Fourier synthesis and refined isotropically. All other hydrogen atoms were included at calculated positions with fixed thermal parameters. All non-hydrogen atoms were refined anisotropically.[8] The crystal of **5** contain large voids, filled with disordered solvent molecules. The size of the voids are 508 Å^3. Their contribution to the structure factors was secured by back-Fourier transformation using the SQUEEZE routine of the program PLATON[9] resulting in 223 electrons/unit cell. MERCURY[10] was used for structure representations.

Crystal Data for **3**: $C_{32}H_{30}N_8$, Mr = 526.64 gmol^{-1}, colorless prism, size 0.108 x 0.104 x 0.100 mm^3, monoclinic, space group P 2_1/c, a = 18.1967(9), b = 8.3459(4), c = 9.2307(5) Å, β = 100.489(3)°, V = 1378.42(12) Å^3 , T= -140 °C, Z = 2, $\rho_{calcd.}$ = 1.269 gcm^{-3}, μ (Mo-K_α) = .79 cm^{-1}, multi-scan,

transmin: 0.6672, transmax: 0.7456, F(000) = 556, 14528 reflections in h(-23/23), k(-10/10), l(-11/11), measured in the range 2.28° ≤ Θ ≤ 27.48°, completeness Θ_{max} = 99.7%, 3138 independent reflections, R_{int} = 0.0522, 2610 reflections with $F_o > 4\sigma(F_o)$, 188 parameters, 0 restraints, $R1_{obs}$ = 0.0528, wR^2_{obs} = 0.1115, $R1_{all}$ = 0.0665, wR^2_{all} = 0.1184, GOOF = 1.069, largest difference peak and hole: 0.279 / -0.210 e $Å^{-3}$.

Crystal Data for **5** $C_{56}H_{50}ClIrN_{10}$[*], Mr = 1090.71 $gmol^{-1}$[*], yellow prism, size 0.088 x 0.084 x 0.082 mm^3, triclinic, space group P ī, a = 8.7392(2), b = 18.7132(4), c = 20.0069(5) Å, α = 114.479(1), β = 96.848(1), γ = 99.180(1)°, V = 2876.43(12) $Å^3$, T= -140 °C, Z = 2, $\rho_{calcd.}$ = 1.259 gcm^{-3}[*], μ (Mo-K_α) = 24.09 cm^{-1}[*], multi-scan, transmin: 0.6201, transmax: 0.7456, F(000) = 1100, 40334 reflections in h(-11/11), k(-24/24), l(-25/25), measured in the range 2.458° ≤ Θ ≤ 27.485°, completeness Θ_{max} = 99.4%, 13121 independent reflections, R_{int} = 0.0229, 12558 reflections with $F_o > 4\sigma(F_o)$, 621 parameters, 0 restraints, $R1_{obs}$ = 0.0280, wR^2_{obs} = 0.0754, $R1_{all}$ = 0.0300, wR^2_{all} = 0.0767, GOOF = 1.163, largest difference peak and hole: 1.360 / -1.744 e $Å^{-3}$.

[*] derived parameters do not contain the contribution of the disordered solvent.

Crystallographic data (excluding structure factors) has been deposited with the Cambridge Crystallographic Data Centre as supplementary publication CCDC-1962576 for **3**, and CCDC-1962577 for **5**. Copies of the data can be obtained free of charge on application to CCDC, 12 Union Road, Cambridge CB2 1EZ, UK [E- mail: deposit@ccdc.cam.ac.uk].

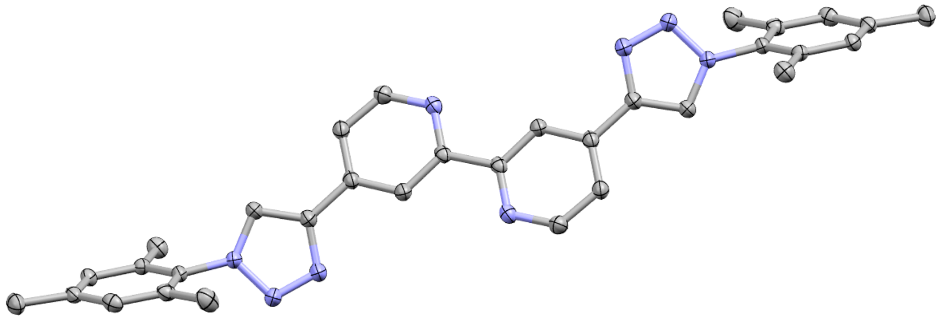

*Figure S9. Molecular structure of receptor **3** (thermal ellipsoids at 50% probability, hydrogens omitted for clarity). Grey, carbon; light blue, nitrogen.*

6. DFT calculations

The theoretical calculations based on density functional theory (DFT) were performed with the Gaussian16 program package (Version A.03).[11] The standard B3LYP functional was selected including Grimme's empirical dispersion correction (GD3) to account for dispersion effects.[12, 13] The 6-31G* basis set were used for all atoms (C,

H, N, Cl) except for I and Ir, which were described by an effective core potential and the associated orbitals (mwb46 and mwb60, respectively). All calculation were performed in the gas phase. The corresponding geometries of the singlet ground states were optimized from reasonable initial estimates. In cases of difficult SCF convergence, additional quadratic (qc) or extra quadratic (xqc) functions were used. The true nature of all minima structures was confirmed by vibrational analysis showing no imaginary frequencies. The graphical visualizations of the three-dimensional representations were generated by GaussView6.0.16.

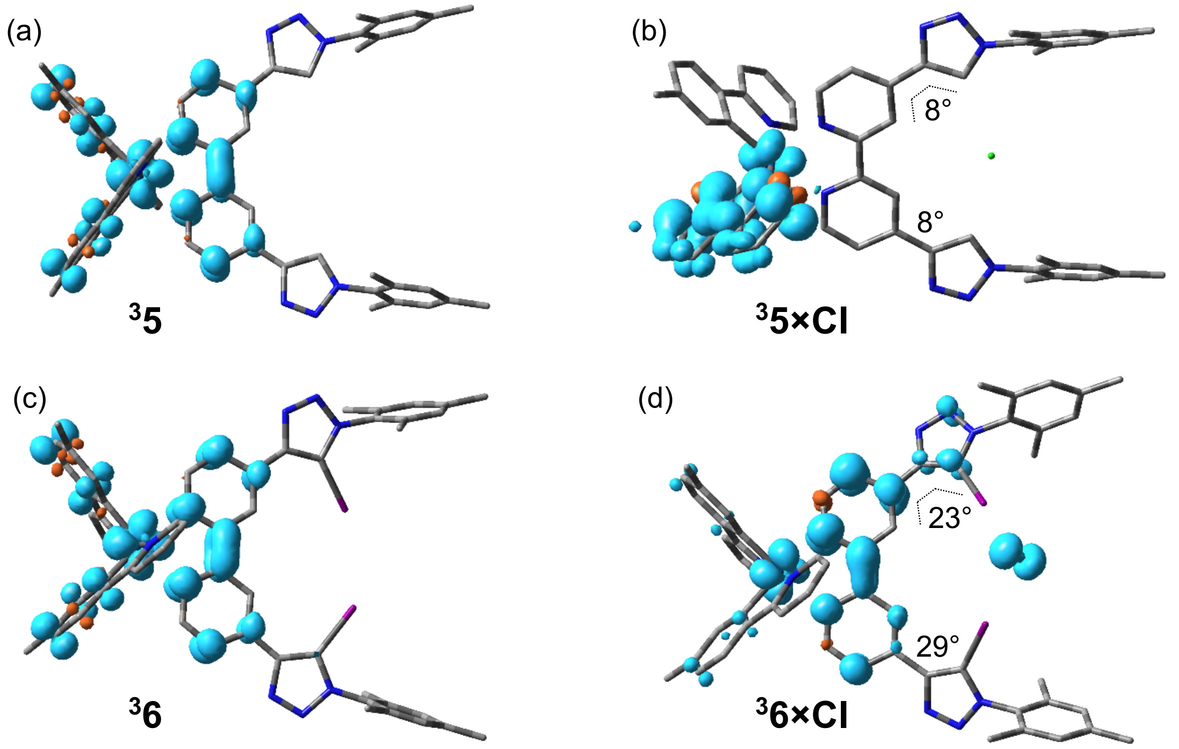

*Figure S10. Spin density plots (isovalue drawn at 0.004) of the triplet states: (a) HB sensor **5**, (b) **5×Cl**, (c) free XB sensor **6** and (d) **6×Cl**. Note the different extent of co-planarity between the triazole and pyridine rings defined by interannular dihedral angle (HB: 8 and 8° vs. XB: 22 and 29°), leading to more pronounced LUMO destabilization for the HB vs. XB systems in accordance to the blue-shifted emission and different spin localization.*

Table S2. Thermodynamic data from DFT calculations.

Receptor	State	Electronic energy (EE) [hartree]E	Zero-point energy (ZPE) [hartree]		Energy (EE+ZPE) [hartree]	ΔSCF energy [eV]	ΔE upon anion binding [eV]
**5**	s0	-2816.04	0.944977		-2815.10		
	t1	-2815.96	0.942112		-2815.02	2.189	
**5×Cl**	s0	-3276.45	0.947334		-3275.50		
	t1	-3276.36	0.943367		-3275.41	2.486	+0.297
**6**	s0	-2837.66	0.923989		-2836.74		
	t1	-2837.58	0.921192		-2836.66	2.163	
**6×Cl**	s0	-3298.05	0.924607		-3297.13		
	t1	-3297.96	0.920581		-3297.04	2.414	+0.251

7. NMR data

The NMR titrations were carried out using CD_3CN and CD_2Cl_2 as solvent. The stock solutions were prepared as described for the emission titrations without the subsequent dilution step. Instead, the host solution (0.5 mL) was transferred into a 5 mm NMR tube. Next, the guest solution was added into the NMR tube by the help of Hamilton syringes and the data was acquired. The titration was then continued by the next addition and so on.

Table S 3. Concentrations of host and guest solutions used for ^{1}H NMR titrations.

Guest	Guest	$[H]_0$	$[G]_0$	Method
**4**	TBACl	2.1×10^{-3} M	4.0×10^{-2} M	^{1}H NMR
**5**	TBACl	5.2×10^{-6} M	5.0×10^{-3} M	^{1}H NMR

7.1. NMR titration of free XB sensor 4 with TBABr

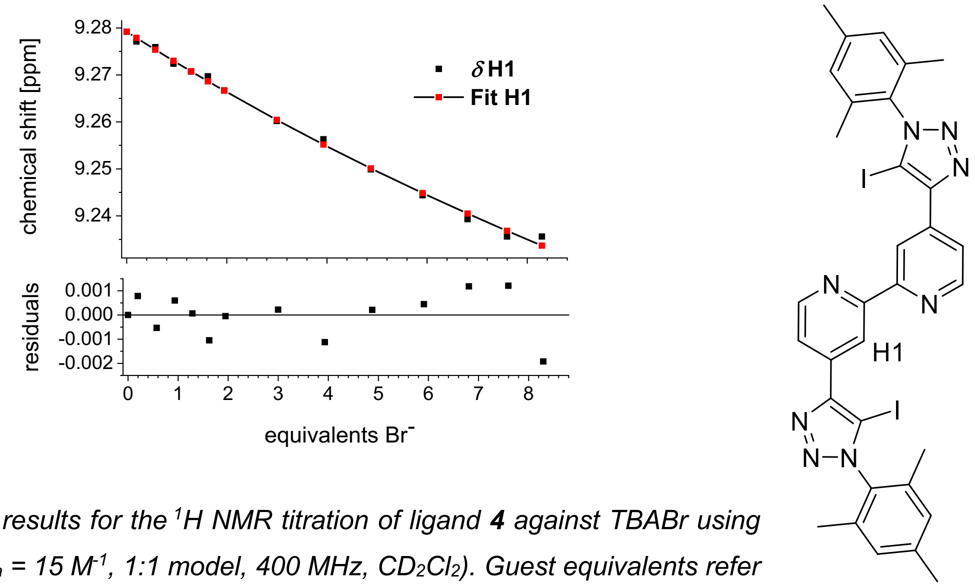

*Figure S11. Fit results for the ^{1}H NMR titration of ligand **4** against TBABr using the H1 peak (K_a = 15 M^{-1}, 1:1 model, 400 MHz, CD_2Cl_2). Guest equivalents refer to the sensor concentration ($[H]_0$ = 2.1 × 10^{-3}).*

7.2. NMR titration of 6

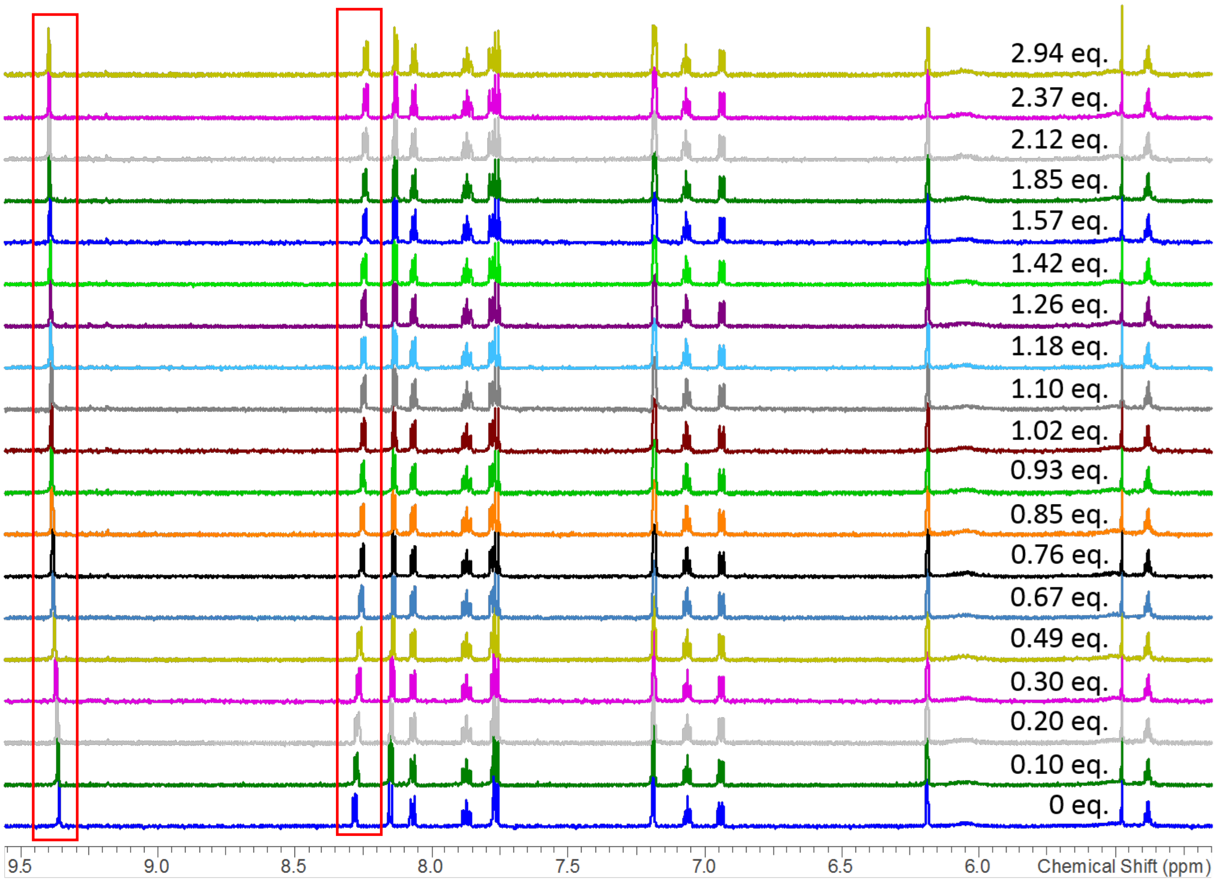

*Figure S12. Aromatic region of the ^{1}H NMR spectra from the titration experiment with **6** against TBACl (600 MHz, CD_3CN). Red box indicated systematic shifts of characteristic proton signals. Guest equivalents refer to the sensor concentration ($[H]_0$ = 4.9 × 10^{-5}).*

7.3. NMR evaluation of XB sensor 6 with TBACl using a 1:1 model

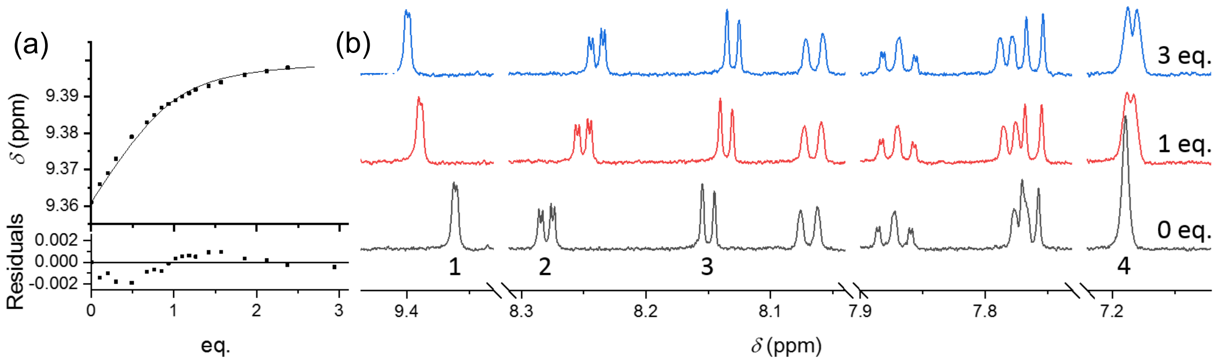

Figure S13. (a) Fit results using a 1:1 model according to the H1 peak (K_a = 1.6 × 10^5 M^{-1} (13%)); (b) Selected spectra from the aromatic region of the ^1H NMR spectra of the XB sensor ***6*** *during ^1H NMR titration (600 MHz, CD_3CN) with 0, 1 and 3 eq. of TBACl. Note the systematic residuals indicating additional processes, e.g. a 2:1 contribution (see manuscript for details). Guest equivalents refer to the sensor concentration ($[H]_0$ = 4.9 × 10^{-5}).*

8. Optical data

All emission titrations were carried out using CH_3CN as solvent. To minimize dilution effects resulting from changes of the host concentration, it was kept constant during the titrations. The host stock solution was prepared by weighting the material as well as the solvent for very accurate concentrations. For the guest stock solution, the calculated mass was dissolved in host stock solution, since the density of the host solution is unknown. This adds a systematical error to the fabrication of the guest solution. While this did not affect the lower concentrated guest solutions for sensor **6**, with higher salt masses the volume changed in a slightly observable manner, when dissolving the salts in the host solution. Subsequently, both solutions were diluted by the factor 100 to reach the final concentrations. 1 mL of the final host solution was transferred to an emission quartz glass cuvette (d = 10 mm). The guest solution was added directly into the cuvette by the help of Hamilton syringes during the titration. All titrations were performed using a Jasco FP-8300 instrument (5 nm band width (ex), 10 nm band width (em), 1 s response, medium sensitivity, 1 nm data pitch, three cycles) spectrometer. The binding isotherms were obtained by fitting the intensity observed at a wavelength against the equivalents of added guest.

Table S 4. Concentrations of host and guest solutions used for ^{1}H NMR titrations.

Host	Guest	$[H]_0$	$[G]_0$	Method
**5**	TBABr	5.1×10^{-6} M	5.0×10^{-3} M	Emission
**5**	TBAOAc	5.3×10^{-6} M	5.0×10^{-3} M	Emission
**6**	TBACl	5.1×10^{-6} M	5.0×10^{-4} M	Emission
**6**	TBABr	5.1×10^{-6} M	5.0×10^{-4} M	Emission
**6**	TBAOAc	3.1×10^{-6} M	1.9×10^{-4} M	Emission
**6**	$TBAPF_6$	5.0×10^{-6} M	4.9×10^{-3} M	Emission
**7**	TBACl	5.3×10^{-6} M	4.9×10^{-3} M	Emission

The quantum yield of both sensors in the same solvent ($n = n_{ref}$) was determined according to equation (1).[14]

$$\Phi_{em} = \frac{A_{ref}}{A} \times \frac{I}{I_{ref}} \times \Phi_{ref} \qquad \textbf{(1)}$$

For that purpose, solutions of the sensors and $[Ru(bpy)_3](PF_6)_2$ in CH_3CN were prepared analogous to the emission titrations and emission spectra were recorded using the same parameters. The calculations were performed using 1.8% as value for the quantum yield of the reference (Φ_{ref}) in air saturated CH_3CN.[15] UV/vis data used for these calculations is shown in Figure S14.

Table S5. Overview over the experimental parameters for the determination of the quantum yield.

**Receptor**	**Host concentration [M]**	**Emission maximum [nm]**	**Quantum yield[a] [%]**
**5**	4.98×10^{-6}	609	2.6
**6**	4.96×10^{-6}	616	2.5
$[Ru(bpy)_3](PF_6)_2$	4.90×10^{-6}	607	1.8[b]

a) Excitation at 400 nm, recording the intensity at the emission maximum. b) Reference value of $[Ru(bpy)_3](PF_6)_2$ in air-saturated CH_3CN taken from ref.[15].

8.1. Absorption and emission spectra

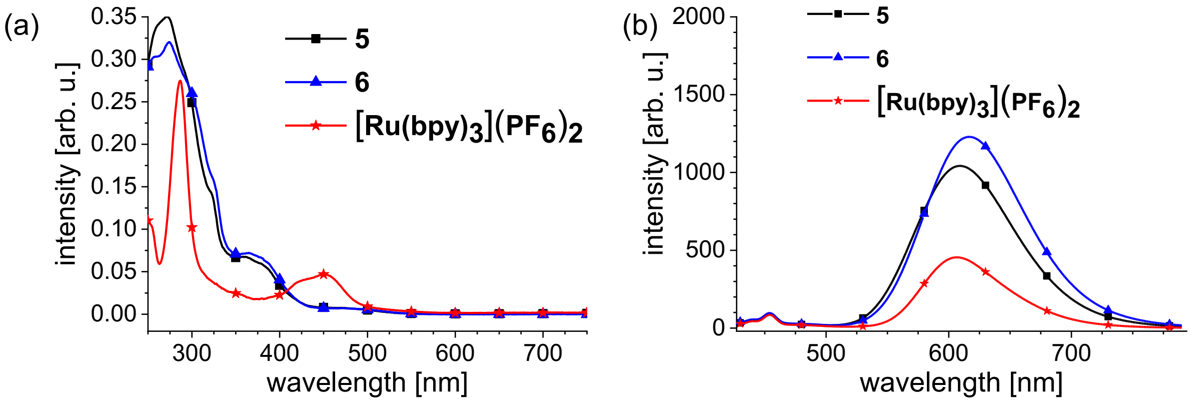

*Figure S14. (a) UV/vis spectra of the sensors **5**, **6** and [Ru(bpy)$_3$](PF$_6$)$_2$ in CH$_3$CN. (b) Corresponding emission spectra of the sensors **5**, **6** and [Ru(bpy)$_3$](PF$_6$)$_2$ in CH$_3$CN (λ_{exc} = 400 nm).*

8.2. Reference titration experiments

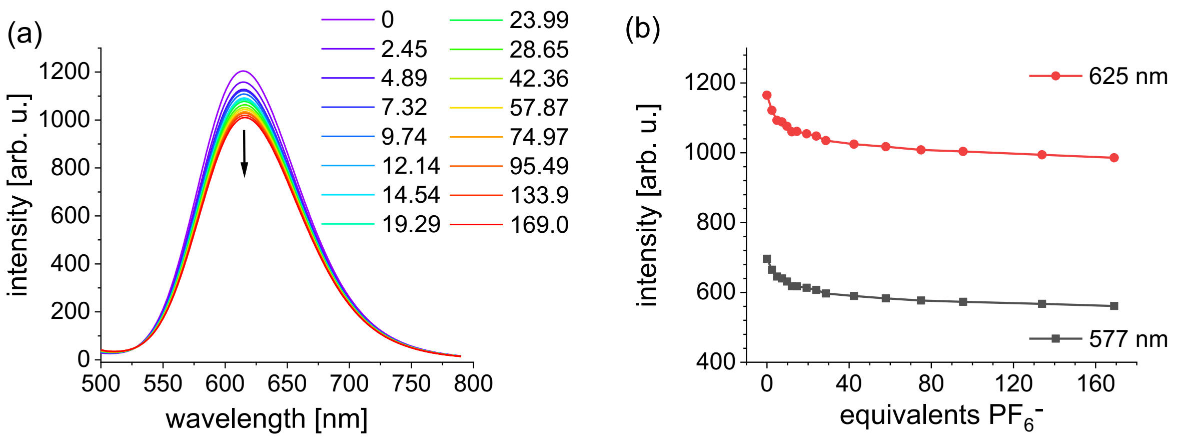

*Figure S15. (a) Spectra of the emission titration of XB sensor **6** against TBAPF$_6$ measured in CH$_3$CN (5.0 × 10^{-6} M). Note the preserved emission maxima without spectral shifts accompanied with continuous decreased intensity. (b) Selected emission wavelengths. Note that concentration profile of each wavelength was used to correct the corresponding emission wavelength data of the HB sensor **5** and XB sensor **6**. Guest equivalents refer to the sensor concentration.*

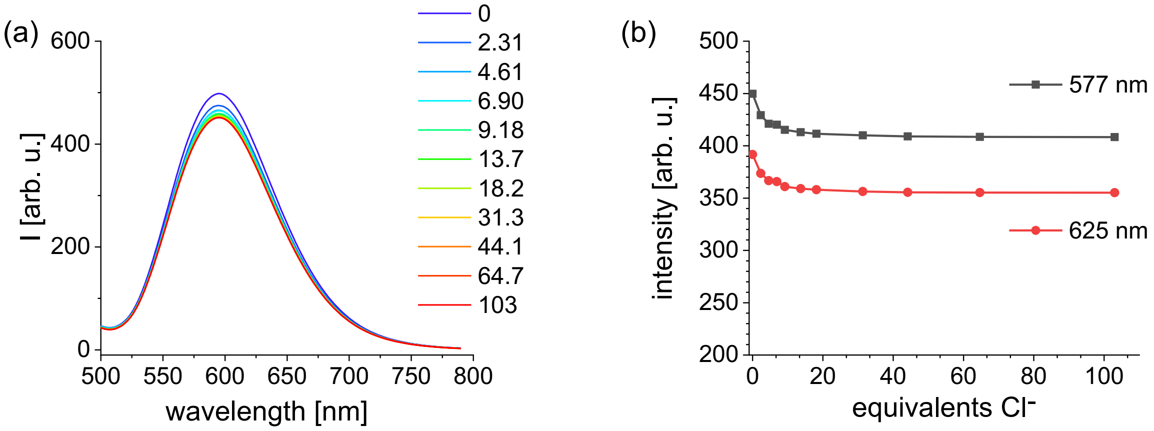

Figure S16. (a) Spectra of the emission titration of reference sensor ***7*** *against TBACl measured in CH_3CN (5.3×10^{-6} M). Note the preserved emission maxima without spectral shifts accompanied with decreased intensity within the first 20 equivalents (the levelling > 20 equivalents may be attributed to weak Cl^- binding). (b) Selected emission wavelengths. Guest equivalents refer to the sensor concentration.*

8.3. Emission correction procedure

The raw emission data was corrected according to the following procedure. The decrease of the intensity profile was derived from the titration data of XB sensor **6** with PF_6^- (Figure S15), *i.e.*, the reference profile was interpolated for the used equivalents of a measurements, scaled to the same initial intensity and subtracted to yield the corrected intensity changes ($\Delta I_{corrected}$). Note that the arbitrary scaling account for the different intrinsic quantum yields (*e.g.* **5** vs. **6**) but has no effect on the analysis of the binding behavior analysis using the BindFit program. This simple and consistent correction was applied in the following, which enabled the analysis of severe overlapping effects (higher intensity due to binding with respect to decrease due to "ionic strength"). Notably, the characteristic and monotonous wavelength shifts (blue-shift) corroborates the reliability of the correction scheme.

8.4. Emission titration of HB sensor 5

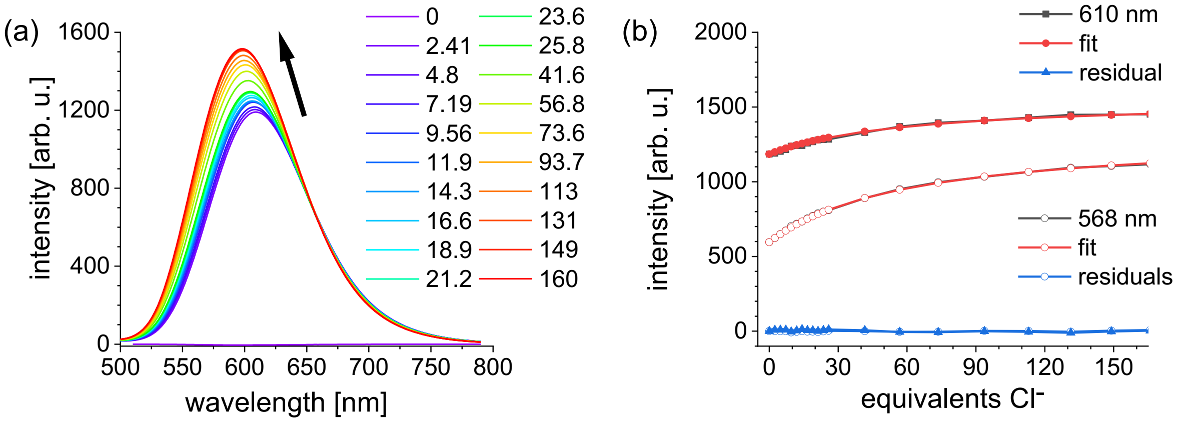

*Figure S17. (a) Emission spectra taken during the titration of HB sensor **5** against TBACl measured in CH_3CN (5.2 × 10^{-6} M). Note that an hypsochromic shift was observed while the intensity increased (arrow). (b) Exemplary fits at 610 nm (filled rectangles) and 568 nm (open circles) of raw data (black), fit result from 1:1 model (red) and residual (blue). Guest equivalents refer to the sensor concentration.*

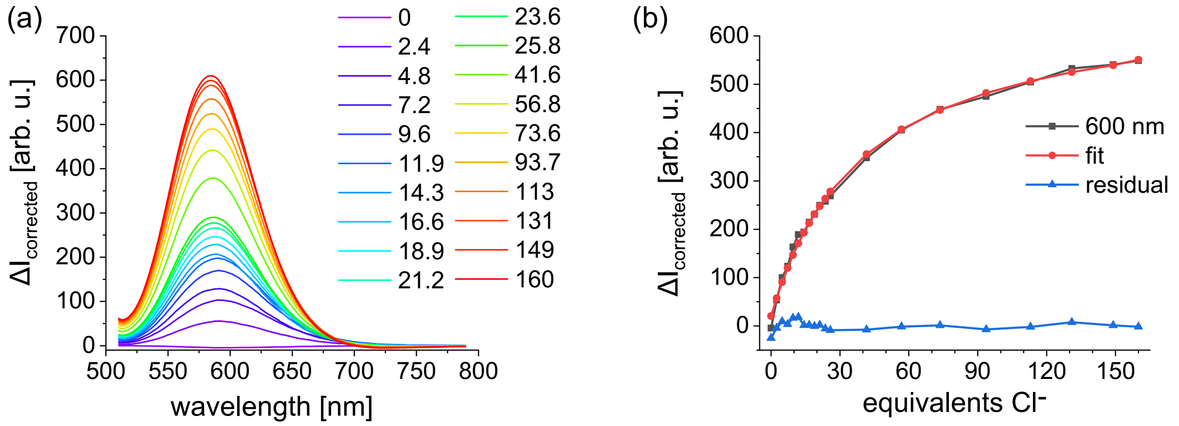

*Figure S18. (a) Spectral changes of corrected emission data ($\Delta I_{corrected}$) of HB sensor **5** with TBACl. (b) Representative trace (600 nm) of corrected data (black), fit result from 1:1 model (red) and residual (blue). See Section 8 for details on emission correction. Guest equivalents refer to the sensor concentration ($[H]_0$ = 5.2 × 10^{-6}).*

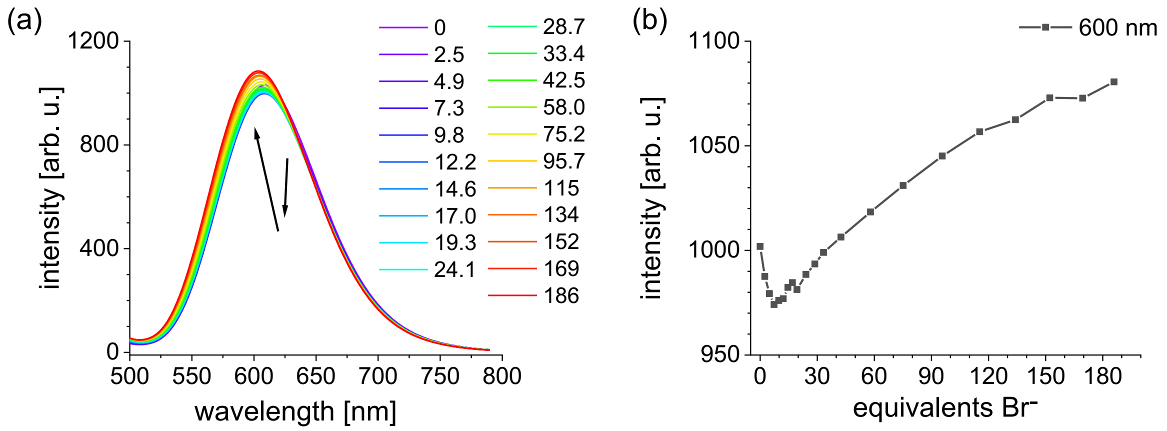

*Figure S19. (a) Emission spectra taken during the titration of HB sensor **5** against TBABr measured in CH_3CN (5.1×10^{-6} M). (b) Exemplary trace at 600 nm. Note that an hypsochromic shift was observed and the intensity increased, after an decrease in the initial stage (>10 equivalents). Guest equivalents refer to the sensor concentration.*

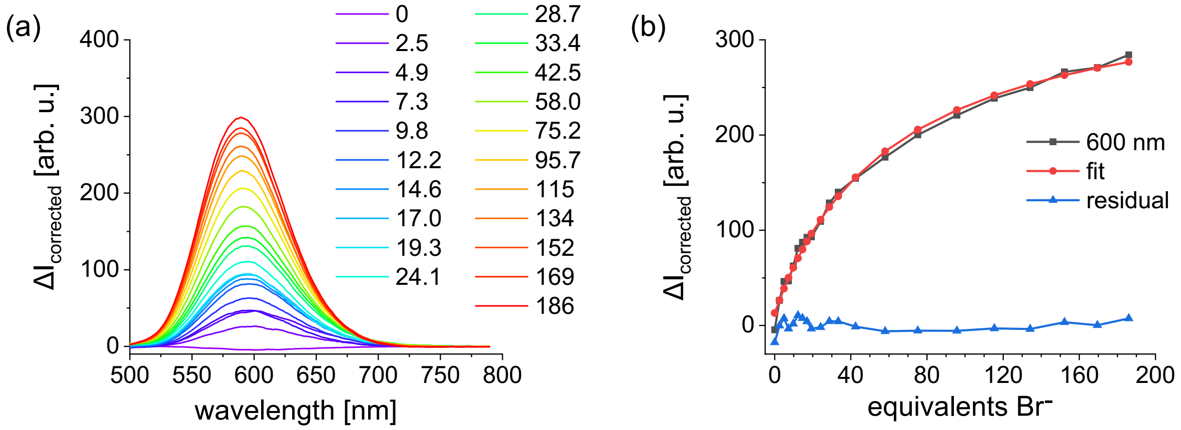

*Figure S20. (a) Spectral changes of corrected emission data ($\Delta I_{corrected}$) of HB sensor **5** with TBABr. (b) Representative trace (600 nm) of corrected data (black), fit result from 1:1 model (red) and residual (blue). See Section 8 for details on emission correction. Guest equivalents refer to the sensor concentration ($[H]_0 = 5.1 \times 10^{-6}$).*

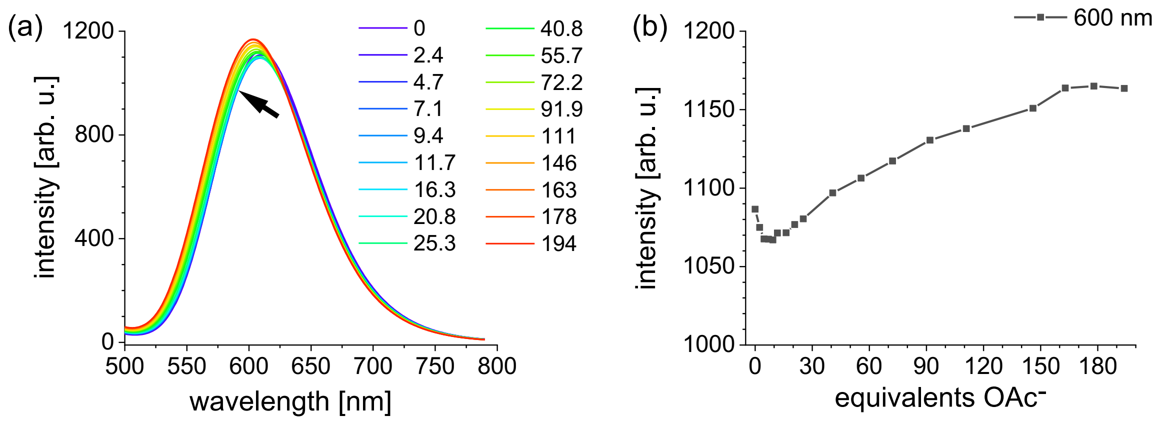

*Figure S21. (a) Emission spectra taken during the titration of HB sensor **5** against TBAOAc measured in CH_3CN (5.3×10^{-6} M). (b) Exemplary trace at 600 nm. Note that an hypsochromic shift was observed and the intensity increased, after an decrease in the initial stage (>10 equivalents). Guest equivalents refer to the sensor concentration.*

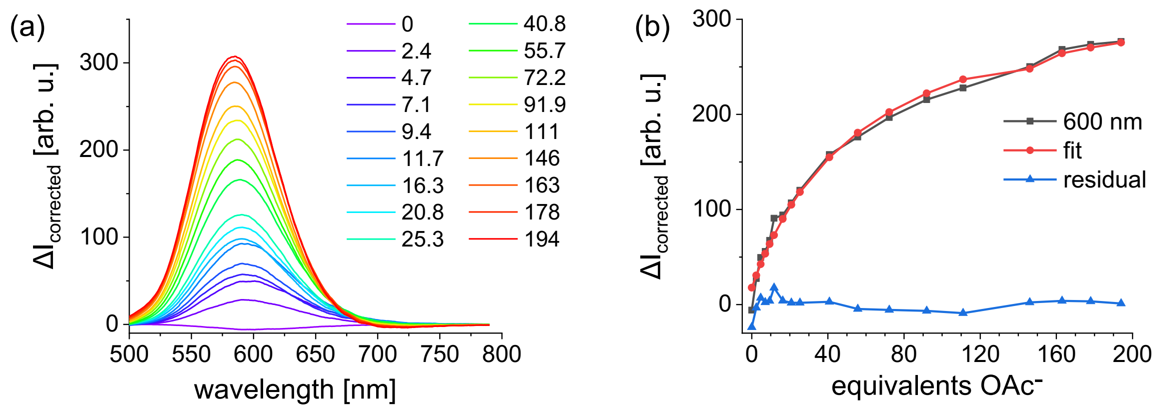

*Figure S22. (a) Spectral changes of corrected emission data ($\Delta I_{corrected}$) of HB sensor **5** with TBAOAc. (b) Representative trace (600 nm) of corrected data (black), fit result from 1:1 model (red) and residual (blue). See Section 8 for details on emission correction. Guest equivalents refer to the sensor concentration ($[H]_0 = 5.3 \times 10^{-6}$).*

8.5. Emission titration of XB Sensor 6

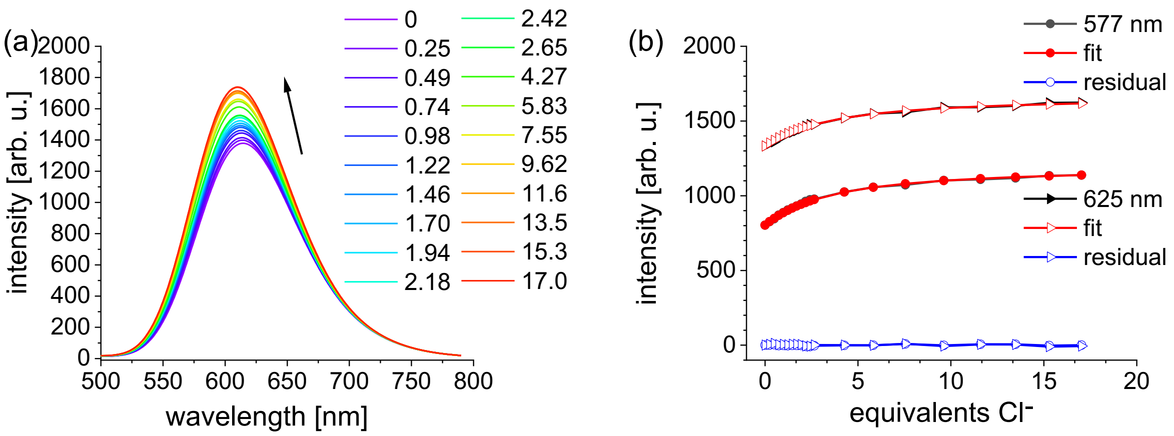

*Figure S23. (a) Spectra of the emission titration of XB sensor **6** against TBACl measured in CH_3CN (5.1×10^{-6} M). Note that an hypsochromic shift was observed and the intensity increased. (b) Exemplary fit results for 1:1 model of the emission titration of sensor **5** against TBACl. Note, that the global fit yields a value $K_a^{uncorrected} = 6.8 \times 10^4$ M (0.3%) which is similar to those obtained from corrected data (vide infra). Guest equivalents refer to the sensor concentration.*

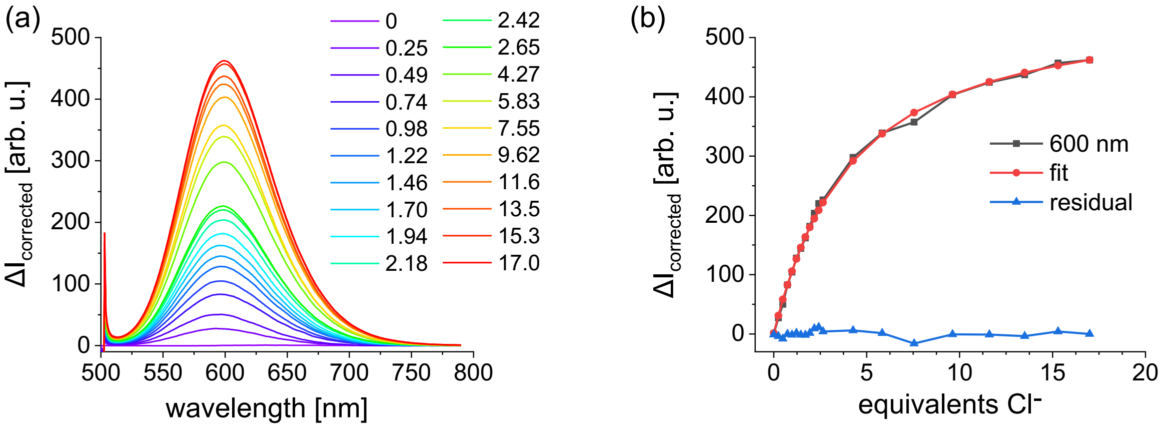

*Figure S24. Spectral changes of corrected emission data ($\Delta I_{corrected}$) of XB sensor **6** with TBACl. (b) Representative trace (600 nm) of corrected data (black), fit result from 1:1 model (red) and residual (blue). See Section 8 for details on emission correction. Guest equivalents refer to the sensor concentration ($[H]_0 = 5.1 \times 10^{-6}$).*

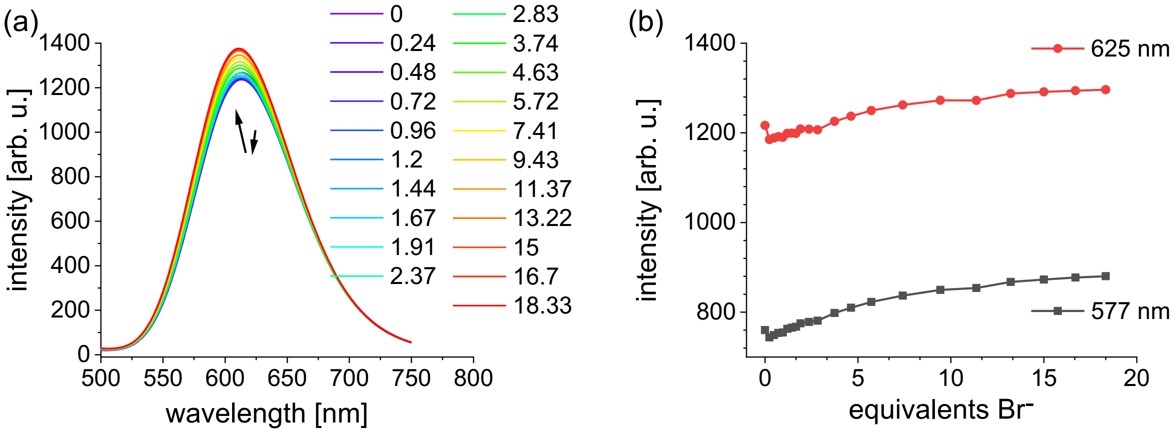

*Figure S25. (a) Emission spectra taken during the titration of XB sensor **6** against TBABr measured in CH_3CN (5.1×10^{-6} M). (b) Exemplary traces at 577 nm and 625 nm. Note that an hypsochromic shift was observed and the intensity increased, after an decrease in the initial stage (> 0.7 equivalents). Guest equivalents refer to the sensor concentration.*

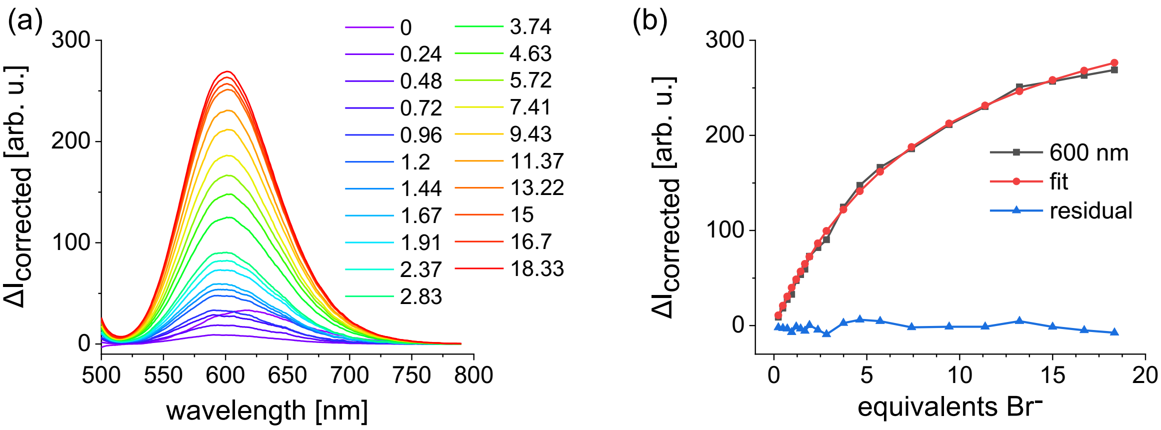

*Figure S26. (a) Spectral changes of corrected emission data ($\Delta I_{corrected}$) of XB sensor **6** with TBABr. (b) Representative trace (600 nm) of corrected data (black), fit result from 1:1 model (red) and residual (blue). See Section 8 for details on emission correction (1st data point omitted in fit). Guest equivalents refer to the sensor concentration ($[H]_0 = 5.1 \times 10^{-6}$).*

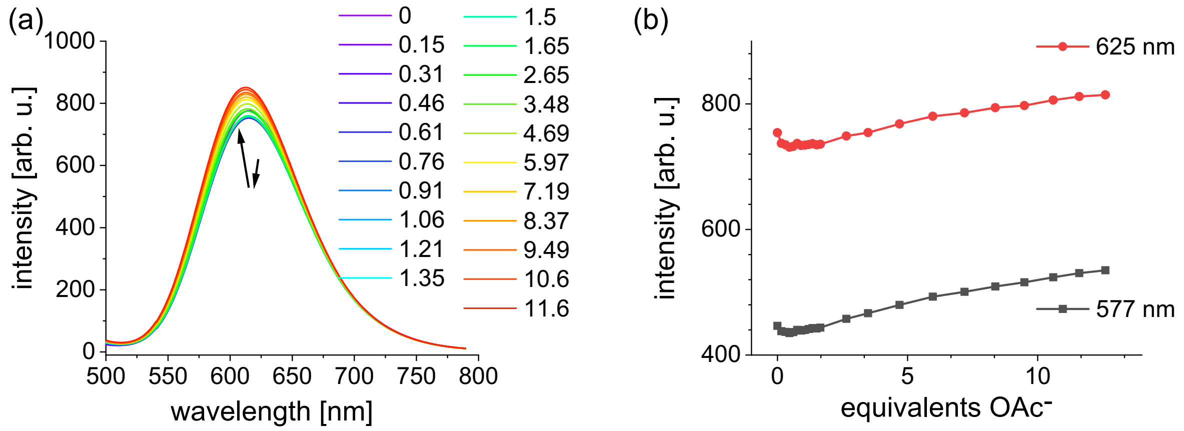

*Figure S27. (a) Emission spectra taken during the titration of XB sensor **6** against TBAOAc measured in CH_3CN (3.1×10^{-6} M). (b) Exemplary traces at 577 nm and 625 nm. Note that an hypsochromic shift was observed and the intensity increased, after an decrease in the initial stage (> 0.7 equivalents). Guest equivalents refer to the sensor concentration.*

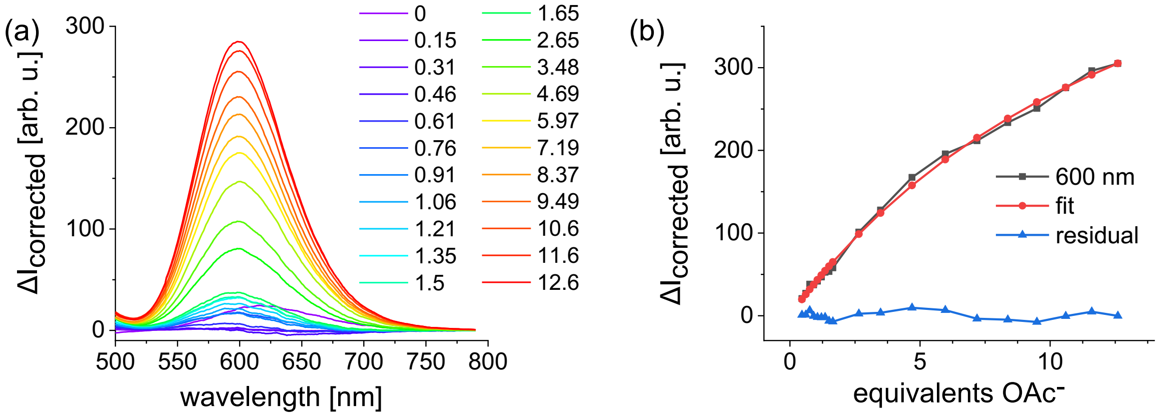

*Figure S28. (a) Spectral changes of corrected emission data ($I_{corrected}$-I_0) of XB sensor **6** with TBAOAc. (b) Representative trace (600 nm) of corrected data (black), fit result from 1:1 model (red) and residual (blue). See Section 8 for details on emission correction (first four data points omitted in fit). Guest equivalents refer to the sensor concentration ($[H]_0 = 3.1 \times 10^{-6}$).*

8.6. Limit of detection

For the determination of the limit of detection (LOD), the emission intensity changes (ΔI) at the maximum wavelength (600 nm) was evaluated. The experimental fluctuations was determined by measuring a blank sample of pure solvent (Figure S29), giving a standard deviation at 600 nm of 0.08 units. The LOD was then calculated by interpolating to the concentration that corresponds to three times the standard deviation of the blank.[16] As a more practical measure for the anion detection under the applied experimental conditions, the SD of the residuals from the fit at 600 nm was taken, which is approximately one order of magnitude higher than the threefold SD of the blank.

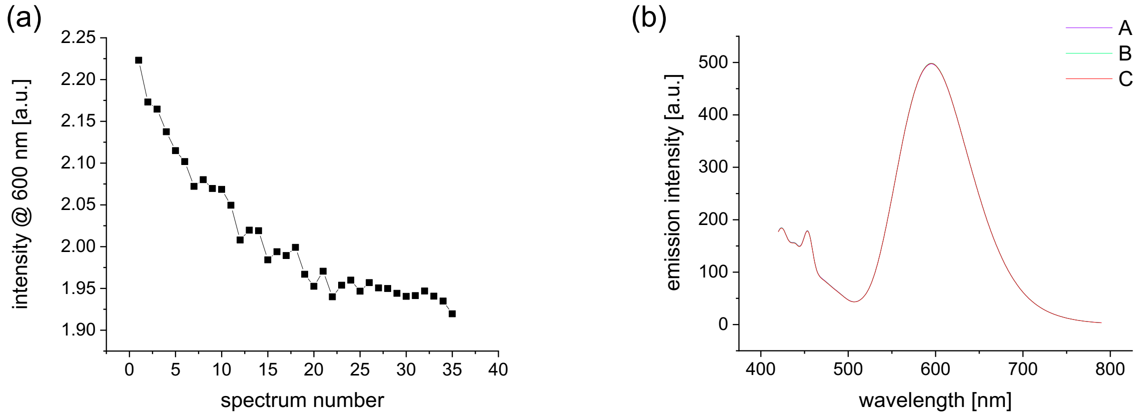

Figure S29. (a) Background emission spectra of blank sample recorded at 600 nm illustrating the experimental fluctuations of consecutive scans. Drift of intensity assigned to lamp intensity changes. Mean values and standard deviation (SD) of full data set amounts 2.01±0.08, the last 10 data points 1.95±0.01. Note that the conservative upper bound (SD=0.08) was used to estimate the limit of detection (LOD). (b) Three consecutive emission spectra of reference complex 7 illustrating baseline stability and negligible changes in the recorded emission spectra in the relevant ^{3}MLCT region (500 to 700 nm).

9. References

1. J.-L. Wang, C. Wang, K. E. deKrafft and W. Lin, *ACS Catalysis*, 2012, **2**, 417-424.
2. F. Kloss, U. Kohn, B. O. Jahn, M. D. Hager, H. Goerls and U. S. Schubert, *Chem. Asian J.*, 2011, **6**, 2816-2824.
3. S. Sprouse, K. A. King, P. J. Spellane and R. J. Watts, *J. Am. Chem. Soc.*, 1984, **106**, 6647-6653.
4. A. Winter, C. Ulbricht, E. Holder, N. Risch and U. S. Schubert, *Aust. J. Chem.*, 2006, **59**, 773-782.
5. B. V. Nonius, *Journal*, 1998.
6. Z. Otwinowski and W. Minor, in *Methods Enzymol.*, ed. Charles W. Carter, Jr., Academic Press, 1997, vol. 276, pp. 307-326.
7. L. Krause, R. Herbst-Irmer, G. M. Sheldrick and D. Stalke, *J. Appl. Crystallogr.*, 2015, **48**, 3-10.
8. G. M. Sheldrick, *Acta Crystallogr. Sect. A: Found. Crystallogr.*, 2015, **71**, 3-8.
9. A. L. Spek, *Acta Crystallogr. Sect. C: Struct. Chem.*, 2015, **71**, 9-18.
10. C. F. Macrae, P. R. Edgington, P. McCabe, E. Pidcock, G. P. Shields, R. Taylor, M. Towler and J. van De Streek, *J. Appl. Crystallogr.*, 2006, **39**, 453-457.
11. M. J. Frisch, G. W. Trucks, H. B. Schlegel, G. Scuseria, M. A. E.; Robb, J. R. Cheeseman, G. Scalmani, V. Barone, B. Mennucci, G. A. Petersson, H. Nakatsuji, M. Caricato, X. Li, H. P. Hratchian, A. F. Izmaylov, J. Bloino, G. Zheng, J. L. Sonnenberg, M. Hada, M. Ehara, K. Toyota, R. Fukuda, J. Hasegawa, M. Ishida, T. Nakajima, Y. Honda, O. Kitao, H. Nakai, T. Vreven, J. Montgomery, J. A., J. Peralta, F. E.; Ogliaro, M. Bearpark, J. J. Heyd, E. Brothers, K. N. Kudin, V. N. Staroverov, R. Kobayashi, J. Normand, K. Raghavachari, A. Rendell, J. C. Burant, S. S. Iyengar, J. Tomasi, M. Cossi, N. Rega, N. J. Millam, M. Klene, J. E. Knox, J. B. Cross, V. Bakken, C. Adamo, J. Jaramillo, R. Gomperts, R. E. Stratmann, O. Yazyev, A. J. Austin, R. Cammi, C. Pomelli, J. W. Ochterski, R. L. Martin, K. Morokuma, V. G. Zakrzewski, G. A. Voth, P. Salvador, J. J. Dannenberg, S. Dapprich, A. D. Daniels, Ö. Farkas, J. B. Foresman, J. V. Ortiz, J. Cioslowski and D. J. Fox, *Journal*, 2010.
12. A. D. Becke, *J. Chem. Phys.*, 1993, **98**, 5648-5652.
13. C. T. Lee, W. T. Yang and R. G. Parr, *Physical Review B*, 1988, **37**, 785-789.
14. C. A. Parker and W. T. Rees, *Analyst*, 1960, **85**, 587-600.
15. K. Suzuki, A. Kobayashi, S. Kaneko, K. Takehira, T. Yoshihara, H. Ishida, Y. Shiina, S. Oishi and S. Tobita, *Phys. Chem. Chem. Phys.*, 2009, **11**, 9850-9860.
16. T. L. Mako, J. M. Racicot and M. Levine, *Chem. Rev.*, 2019, **119**, 322-477.
